# Disruption of *SorCS2* reveals differences in the regulation of stereociliary bundle formation between hair cell types in the inner ear

**DOI:** 10.1371/journal.pgen.1006692

**Published:** 2017-03-27

**Authors:** Andrew Forge, Ruth R. Taylor, Sally J. Dawson, Michael Lovett, Daniel J. Jagger

**Affiliations:** 1 UCL Ear Institute, University College London, London, United Kingdom; 2 National Heart and Lung Institute, Imperial College London, London, United Kingdom; Emory University School of Medicine, UNITED STATES

## Abstract

Behavioural anomalies suggesting an inner ear disorder were observed in a colony of transgenic mice. Affected animals were profoundly deaf. Severe hair bundle defects were identified in all outer and inner hair cells (OHC, IHC) in the cochlea and in hair cells of vestibular macular organs, but hair cells in cristae were essentially unaffected. Evidence suggested the disorder was likely due to gene disruption by a randomly inserted transgene construct. Whole-genome sequencing identified interruption of the *SorCS2* (Sortilin-related VPS-10 domain containing protein) locus. Real-time-qPCR demonstrated disrupted expression of *SorCS2* RNA in cochlear tissue from affected mice and this was confirmed by SorCS2 immuno-labelling. In all affected hair cells, stereocilia were shorter than normal, but abnormalities of bundle morphology and organisation differed between hair cell types. Bundles on OHC were grossly misshapen with significantly fewer stereocilia than normal. However, stereocilia were organised in rows of increasing height. Bundles on IHC contained significantly more stereocilia than normal with some longer stereocilia towards the centre, or with minimal height differentials. In early postnatal mice, kinocilia (primary cilia) of IHC and of OHC were initially located towards the lateral edge of the hair cell surface but often became surrounded by stereocilia as bundle shape and apical surface contour changed. In macular organs the kinocilium was positioned in the centre of the cell surface throughout maturation. There was disruption of the signalling pathway controlling intrinsic hair cell apical asymmetry. LGN and Gαi3 were largely absent, and atypical Protein Kinase C (aPKC) lost its asymmetric distribution. The results suggest that SorCS2 plays a role upstream of the intrinsic polarity pathway and that there are differences between hair cell types in the deployment of the machinery that generates a precisely organised hair bundle.

## Author summary

Sensory “hair” cells in the inner ear derive their name from an organised bundle of mechano-sensory “stereocilia” on their apical surface. The finger-like stereocilia are composed of actin filaments and increase in height staircase-like in one direction across the cell surface, a system which provides a defined polarity across the sensory epithelium. The establishment of hair bundle polarity and orientation is a highly regulated process involving several signalling pathways. There are subtle differences in bundle morphology between hair cell subtypes, suggesting local variations exist within these regulatory pathways. In a transgenic mouse colony we found profoundly deaf animals displaying severe hair bundle abnormalities on all auditory hair cells. Hair cells in the gravity sensing tissues of the balance system also had abnormal bundles, but there was no effect on bundles of hair cells sensing rotation. Hair bundle abnormalities coincided with mis- expression of proteins of an intrinsic apical asymmetry pathway. These defects were associated with disruption of the *SorCS2* gene, and consequent mis-expression of the coded transmembrane receptor in the inner ear. The results suggest that SorCS2 regulates hair cell development, and that there are previously unrecognised differences in the way subtypes of hair cells form their stereociliary bundles.

## Introduction

The sensory “hair” cells of the hearing and balance (vestibular) organs in the inner ears of vertebrates convert movements, initiated by sound waves in the cochlea or by translational or rotational motion in the vestibular system, into electrical signals. Fundamental to their function is the organised bundle of stiff, erect projections from their apical (luminal) surface [1] from which hair cells derive their name. This “hair bundle” is formed of stereocilia, microvillus-like protrusions formed of closely packed actin filaments [2, 3], organised in rows that increase in height in one particular direction across the apical surface of the cell. Eccentrically positioned behind the row of longest stereocilia is a single specialised true cilium known as the kinocilium, except uniquely in the mature auditory epithelium in mammals, the organ of Corti, where a kinocilium is present as hair cells first differentiate but subsequently retracts to leave only the basal body in the apical cytoplasm [4]. The asymmetry deriving from the increasing height of stereocilia in one particular direction towards the position of the kinocilium/basal body defines a cellular “polarity” which is of fundamental functional significance [1, 5]. Deflections of the stereocilia along the line of polarity, towards and away from the position of the kinocilium/basal body, opens and closes “mechano-transduction” channels [6], initiating hair cell responses.

The polarity of hair-bundles on different hair cells with respect to each other is not random. Hair-bundles show a distinct “orientation”, as defined by the position of the kinocilium/basal body, that is precisely related to that of their immediate neighbours. In the organ of Corti the hair bundles on all hair cells are oriented with the row of longest stereocilia on the side of the bundle towards the outside of the spiralling sensory epithelial strip (the lateral side). In the maculae, flat sheets of sensory epithelium in the utricle and saccule of the vestibular system, all the hair bundles located on one side of a region running along the length of the epithelial sheet are oriented the same way with respect to the periphery and at 180° to those on the other side of that region. In the saddle-shaped cristae of the semi-circular canals, all the hair bundles are oriented in the same direction across the epithelium.

When hair cells first begin to differentiate during embryonic life, around embryonic day (E) 12.5–13.5 in mice, the kinocilium emerges in the centre of the hair cell surface surrounded by nascent stereocilia [7, 8]. Subsequently the kinocilium assumes an eccentric position and stereocilia grow differentially to produce the morphologically polarised and oriented bundle [7, 9, 10]. In the organ of Corti (and the basilar papilla, the auditory epithelium of birds) the length of the longest stereocilia and the number of stereocilia on each hair cell are precisely defined [10–12]. In auditory hair cells, the number of stereocilia that initially emerge exceeds the number ultimately present on the fully differentiated cell; the supernumerary presumptive stereocilia on the inner side (the side of the shorter stereocilia) are retracted into the cell as the remaining ones lengthen [9, 10]. In addition, while the early immature bundles are essentially rounded, mature hair bundles have a characteristic shape, particularly those in the organ of Corti. As the extra stereocilia are withdrawn, hair bundles on cochlear inner hair cells (IHC) become like a wide U-shape or linear and reduced to 2–3 rows (in mice), while bundles on the three rows of outer hair cells (OHC) each consist generally of three rows of stereocilia and assume a characteristic M-shape.

The establishment and maintenance of hair bundle polarity and orientation are thought to be regulated through a number of interacting pathways. Vertebrate homologues of the planar cell polarity (PCP) proteins that are involved in the eccentric positioning of hairs on cells of the wing in *Drosophila*, such as Vangl2, flamingo, frizzled and prickle [13, 14] localise in distinct asymmetric patterns that match hair bundle orientation at the level of the tight-adherens junctional specialisations between a hair cell and the non-sensory supporting cells that surround it [15, 16]. Disruption of the genes that encode these proteins result in mild to moderate disturbance of bundle orientation [15, 16] in a proportion of (mainly) OHC but does not affect cellular polarity—the ranking of stereocilia on individual hair cells. These core PCP proteins are thought to be involved primarily in co-ordinating orientation across the tissue.

Bundle polarity at the cellular level may be regulated through the kinocilium/basal body. Mutations in the genes encoding ciliopathy-associated proteins, ift88 (intraflagellar transport 88) and Kif3a (a component of the kinesin II motor) all of which are involved in maintenance of cilia and which localise in hair cells to the kinocilium or its basal body, result in varying degrees of mis-orientation and/or disturbance of bundle shape in some OHC [17–22]. These observations have led to investigation of proteins that regulate orientation of the mitotic spindle and centrosome positioning during asymmetric cell division [23–25]. Mammalian inscuteable (mInsc), LGN (Leu-Gly-Asn repeat-enriched protein, also known as mammalian Partner of inscuteable [mPins] or G-protein signalling modulator 2 [Gpsm2]), Gαi3 (GTP-binding protein alpha-I subunit 3), aPKC (atypical protein kinase C) and Par3/Par6 (partitioning-defect-3 and 6), which have roles in coupling the centrosome to the cortical cytoskeleton, are expressed in cochlear hair cells. They are asymmetrically distributed across the apical surface of both OHC and IHC during the formation and maturation of the hair bundles in complementary compartments that outline the hair bundle [23, 25]. Ablation of the genes encoding mInsc and LGN and loss or inactivation of Gαi3 all lead to disruptions of varying severity to the shape and orientation of the bundles in a proportion of OHC and some IHC. These “intrinsic polarity pathway proteins” [25] are thought to interact with the cortical cytoskeleton underlying the apical surface plasma membrane to position the kinocilium and shape the bundles of these cochlear hair cells, thereby controlling hair cell apical asymmetry. A similar asymmetric distribution of the intrinsic polarity proteins is also evident in macular hair cells [23] but the effects of their inactivation upon hair bundle morphology and organisation in vestibular sensory epithelia has not been reported. In this paper we demonstrate disturbances of hair bundle morphology more severe than those described for mutations in other genes involved in hair bundle formation, which affect every cochlear IHC, cochlear OHC and vestibular macular hair cell, with different effects on each of these hair cell types, but which does not affect the hair bundles of hair cells in the cristae. This phenotype is associated with the mis-expression or mis-localisation of intrinsic polarity pathway proteins. We also show that the phenotype is associated with a disruption in the gene encoding SorCS2 (Sortilin-related VPS-10 domain containing protein). SorCS2 has not previously been implicated in hair bundle differentiation.

## Results

**Fig 1:**
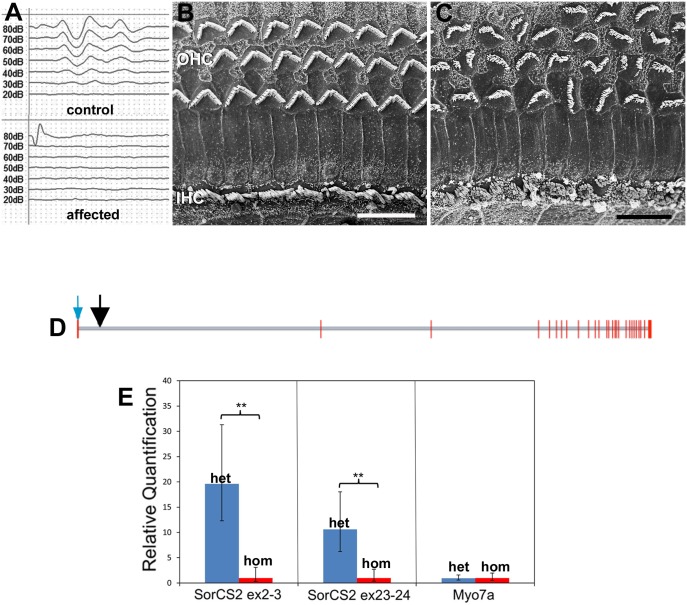
Hearing loss and hair bundle defects are associated with *SorCS2* disruption. **A**. Representative ABRs to click stimuli from a normal mouse (top) and an affected littermate (bottom) mouse at P17. There are no recordable responses from the affected animal, while the normal had an auditory threshold of 20-30db SPL. **B,C.** Organs of Corti from the upper basal-middle cochlear turn of **normal (B)** and **affected (C)** littermate mice at P17. OHC: outer hair cells; IHC inner hair cells. The hair bundles of every OHC and every IHC in the affected animal show severe morphological abnormalities. Scale bars: 10μm. **D. The genomic structure of the SORCS2 gene and the position of the transgene insertion.** The gene spans ~380kb and encodes 27 exons shown as red vertical lines. Introns are to scale and are shown in grey. The transcriptional direction is left to right. The translational start site is denoted by a blue arrow. The position of the transgene insertion is shown by a black arrow ~15kb into intron 1. It occurs between nucleotides 36382157 and 36382308 on mouse chromosome 5 (GRCm38/mm10 genome build) resulting in the deletion of the intervening bases in addition to the transgene insertion. **E. Relative expression levels of mRNA from exon 2–3 and exon 23–24 of *SorCS2* and of control hair cell “marker” gene, that encoding Myo7a, in extracts from the whole inner ear at P1**. Blue bar, heterozygous (het); red bar affected homozygous (hom) littermates. Level of expression of SorCS2 mRNA from 5 of 9 homozygous animals is significantly lower than that of heterozygous littermates. In the other 4 homozygous animals no mRNA from either the two *SorCS2* exons was detected. Levels of expression of mRNA for the control gene, that of Myosin 7a, is the same in both heterozygous and homozygous animals.

The possibility of an inner ear defect arose in a colony of mice being used for studies of c-type natriuretic peptide (CNP) in endothelial cells, in which mice carrying a *cre* construct driven by the promoter for the endothelial specific protein Tie-2 (*Tie2-cre* animals) were being crossed with mice containing floxed *CNP*, both transgenic strains on a C57Bl/6 background [26]. Some animals in this colony exhibited behavioural anomalies including hyperactivity and significant periods hanging from and walking across the cage tops, peculiarities which have been associated with vestibular disturbance [27, 28]. ABR recording, to assess hearing sensitivity, identified a sub-population of animals that were profoundly deaf, with no recordable response to click stimuli of 80dB at the earliest age examined (P15); all other animals had auditory thresholds of between 20 and 30db (Fig 1A). The deaf animals were typically the smallest in the litters, weighing approximately 66% the weight of hearing litter mates (11 affected animal/33 littermates from 5 litters of different ages P15-P35).

Scanning electron microscopy of the organs of Corti of affected animals, at all ages following the normal onset of hearing (P12), revealed morphological anomalies of the hair bundle of every OHC and of every inner hair cell IHC (Fig 1B and 1C) along the entire length of the organ of Corti spiral from base to apex, although defects of OHC, but not IHC, were less severe at the apex (S1 Fig). There was no apparent effect on the length of the organ of Corti or on its width which increased systematically from base to apex (S1 Fig). Measurement of the width of the heads of the inner pillar cells, which separate the row of IHC from the innermost row of OHC, and of the width across OHC region at two defined locations—in the middle of the basal coil and a half turn down from the apical tip—showed no differences in these dimensions (base: ca. 8–9 μm for pillar cell head, and ca. 12–14 μm for OHC region; apex: ca. 14μm and ca. 19μm respectively) between the organs of Corti with abnormal hair bundles and those where the bundles were normal.

### Identifying the genetic disruption

As an initial step to identify the genetic defect in the mouse colony in which the hair bundle anomalies arose, the inner ear phenotypes of all possible genotypes with respect to the presence or absence of the transgenic inserts and in the various combinations were assessed. This revealed that the common feature of all animals that showed bundle anomalies was homozygosity for the *Tie2-cre* insert. The *Tie-2-cre* mice in the colony had been derived from animals in which two copies of the transgene had been inserted randomly into the genome [29]. It was reasoned therefore that the insertion had caused a gene disruption. Consequently, next generation (Illumina HiSeq) whole genome sequencing was applied to identify the site of the disruption.

Total genomic DNA extracted from deaf mice that showed hair bundle anomalies was analysed by Next Gen DNA sequencing. All of the paired end sequence reads that contained any part of the *Tie2-cre* vector were computationally identified. Sequences that contained the internal transgene at both ends were discarded. Of the remaining paired end reads, 21 had reads that at one end contained the transgene vector and at the other end mapped within an Ig Kappa gene. The Ig Kappa family has ~180 closely related members which makes unequivocal mapping difficult. There are no reports of a role for IgK in actin assemblies or similar functions, and immunolabelling for IgK in frozen sections of early postnatal inner ear was confined to capillaries and none was detectable in cells of the sensory epithelium. An additional 13 vector-host paired end sequences mapped to the *SorCS2* gene encoding the Sortilin-Related VPS10 Domain Containing Receptor 2. Specifically, these insertions occurred~15kb into the first intron of *SorCS2* at positions 36382105 to 36382309 on mouse chromosome 5 (NCBI37/MM9) (Fig 1D). Both ends of the insertion were detected in multiple sequence reads. The intervening 204bp of intronic sequences had been deleted and subsequent BLAST analysis of the whole genome DNA sequence (of the homozygous transgenic mouse) confirmed this. SorCS2 is a transmembrane glycoprotein receptor that is a member of the mammalian vacuolar protein sorting 10 protein (VPS 10P) family that have a role in intracellular trafficking of proteins [30], but is also a pro-neurotrophin receptor involved in regulation of actin dynamics associated with neural growth cone collapse [31]. The association of the protein with regulation of actin assemblies therefore suggested disruption of *SorCS2* as a likely cause of the hair bundle anomalies. Evidence for the expression of *SorCS2* in the inner ear of affected and unaffected littermates was therefore sought.

In order to assess whether the transgenic insertion within intron 1 of the *SorCS2* gene affects expression of the major *SorCS2* transcript (Ensembl ID: Sorcs2-001, ENSMUST00000037370.13)

RT-qPCR was performed on cDNA made from whole inner ear tissue removed from P0-P1 animals that were the offspring of crosses between homozygous affected animals and heterozygotes (as identified from the ratios of the phenotypes in their litters). The samples included the cochlea, the vestibular maculae and the cristae. RT-qPCR was performed on tissue from one ear of each animal; the opposite ear was prepared for subsequent phenotypic assessment. RT-qPCR revealed that *SorCS2* was expressed in the inner ear tissues of unaffected animals at this age (Fig 1E). Expression of *SorCS2* varied in affected animals. No expression was detected in 4 out of 9 affected animals. In the remaining 5, expression was much reduced compared to unaffected ones (Fig 1E) with the mean relative level of expression 19 fold lower. Very similar results were obtained using two different expression assays, one spanning exons 2 and 3 of the *SorCS2* gene (i.e. the first two exons immediately following the intronic insertion) and one spanning exons 23 and 24 at the 3’ end of the gene. There was no difference between unaffected and affected animals in the relative expression of the gene encoding the hair cell “marker” protein myosin 7a (Fig 1E).

**Fig 2:**
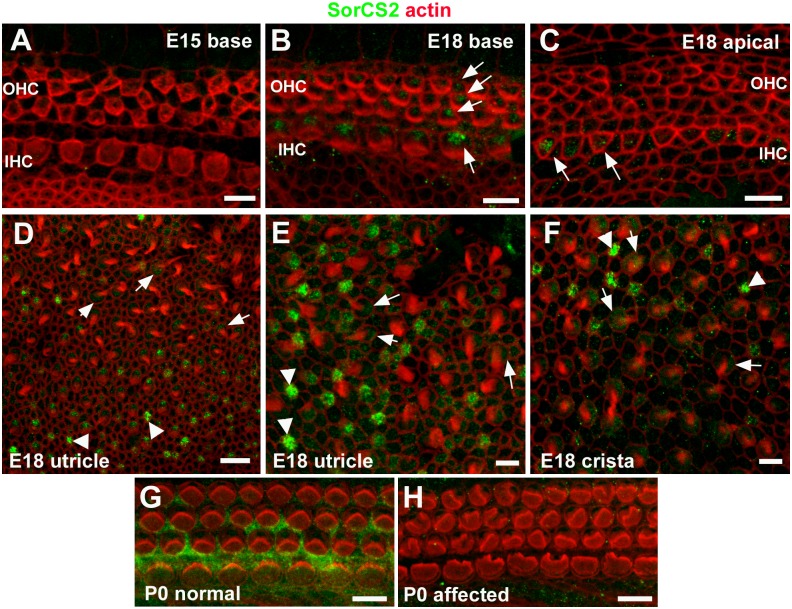
Immuno-labelling for SorCS2 in whole mount preparations of sensory epithelia of the inner ear. In the cochlear basal turn of an E15.5 wild type mouse, immunofluorescence associated with an antibody targeting the extracellular domain of SorCS2 (SorCS2-ED) was not detectable (**A**). At E18, SorCS2 immunofluorescence was localised to inner hair cells (IHC) and outer hair cells (OHC) in the basal turn (**B**, arrows), and to IHC in the apical turn (**C**, arrows). In the utricular macula of the same animal (**D-E**) the SorCS2 antibody localised to presumptive immature hair cells lacking stereociliary bundles (arrowheads) and to hair cells with bundles (arrows). Comparable labelling occurred in hair cells of E18 crista ampullaris (**F**). In the organ of Corti in normal animals at P0 (**G**) the SorCS2 immunofluorescence was localised to hair cells and supporting cells, but it was undetectable in a littermate with affected hair bundles (**H**). Scale bars: A-C, E-H 5 μm; D 10 μm.

Using previously characterised antibodies that target distinct regions of the SorCS2 molecule [32, 33] immuno-labelling of inner ear whole-mount preparations from late embryonic and early postnatal wild-type mice confirmed expression of SorCS2 protein in the organ of Corti, utricular maculae and cristae (Fig 2 and S2 Fig). The expression of SorCS2 was evident in hair cells in all three sensory regions in the embryonic inner ear around the time at which hair bundles are forming (E15-18; Fig 2A–2F). In early postnatal organ of Corti (P0-P1) SorCS2 became increasingly evident in organ of Corti supporting cells (Fig 2G; S2C and S2D Fig). This expression pattern persisted in the mature organ of Corti (P30, S2G Fig). The organ of Corti of unaffected P1 mice expressed relatively higher amounts of SorCS2 in comparison to that of affected littermates (Fig 2G and 2H; S2C–S2F Fig). These results demonstrate (i) that SorCS2 is expressed in all sensory epithelia of the inner ear, and (ii) that disruption of the SorCS2 gene locus results in reduced SorCS2 protein expression.

### Hair bundles in the organ of Corti

#### OHC bundles were severely disrupted

**Fig 3:**
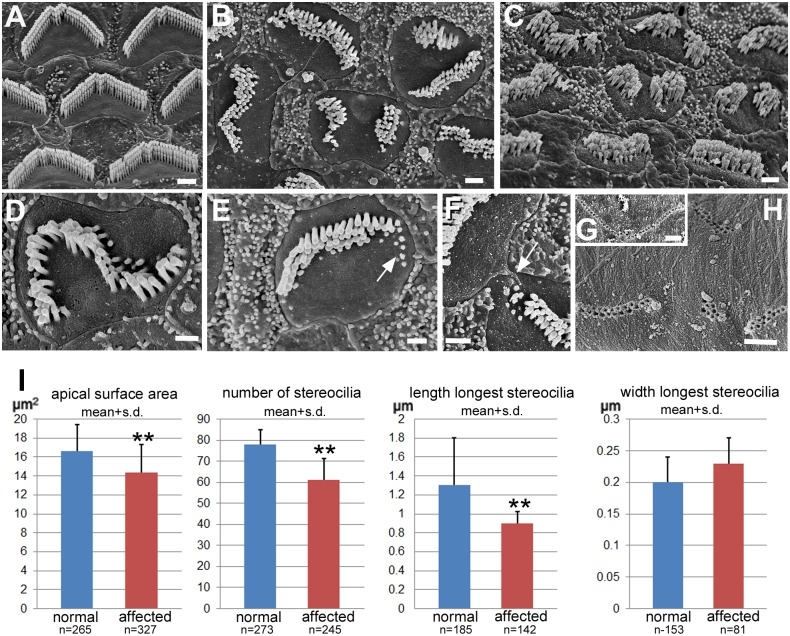
Outer hair cells. Stereociliary bundles and apical surfaces. **A**. **Normal animal at P18.** Basal coil. Stereociliary bundles have a consistent characteristic shape and are entirely surrounded by smooth apical surface membrane. **B-F. Affected animals at P18. B.** Basal coil. Stereociliary bundles in affected animals show a variety of abnormal morphologies but each bundle is entirely surrounded by smooth apical surface membrane. Some bundles are separated completely into two clusters, with smooth apical surface membrane between. The contour of the border of the apical surface of the cell matches the bundle shape: note indent at approximate mid-point of separation between two clusters; and elongated shape of cell surface in orientation matching that of elongated bundle. **C**. Basal coil. Bundles appear shorter than normal (compare with normal in A at same magnification) and are much less regularly organised. **D**. Bundle grossly mis-shapen, but shows stereocilia in 3 rows of increasing height. The contour of the surface membrane matches the shape of the bundle. **E.** Bundle showing apparent shortening/retraction of stereocilia (arrow). **F.** Two OHC in direct contact at their apical surface (arrow). Underside of the tectorial membrane. **G. Normal.** Impressions of the longest stereocilia of the OHC bundle (basal coil) in a single row in the shape of the normal bundle. **H. Affected animal (P18)**. Impressions show that stereocilia of misshapen bundles reach the tectorial membrane but reveal the various morphological peculiarities of the bundles: separate clusters of impressions; and more than one row of impressions from a single bundle indicating mis-regulation of stereocilia height in individual bundles. Scale bars: A-C 1μm; D-F 0.5μm; G,H 1μm. **I.** Histograms displaying means of apical surface area, number of stereocilia and length and width of longest stereocilia on OHCs in normal and affected animals. Each mean was derived from pooled measurements/counts at basal, middle and apical coils in individual cochleae from at least 3 different animals aged P17-P22; n = number of cells assessed in each group. The hair bundles of OHCs in affected animals have significantly fewer and significantly shorter stereocilia than those of normal animals.

Normal OHC bundles in young hearing animals (P15-P35) (Fig 3A) consisted of three rows of stereocilia of ascending height organised in a distinct “M”-shape and with uniform orientation. The bundles in affected littermate animals of the same ages showed a variety of unusual and ill-defined shapes, were often divided into two or three separate clusters of stereocilia and in some bundles there were what appeared to be only stumps of stereocilia, most usually at the outer edges (Fig 3B–3E). There was no obvious bundle orientation definable. However, generally the stereocilia were in three, or sometimes four, rows of ascending height so that even highly mis-shapen bundles showed an asymmetry (Fig 3C–3E). The bundle occupied only a proportion of the apical surface on which there were “bare” areas free of stereocilia (Fig 3B, 3D and 3E) as exists to the front and back of the bundle in normal OHC (Fig 3A). While the shape of the bundle was often highly distorted or even divided into separated clusters, the shape of the apical surface of the OHC conformed to that of the bundle (Fig 3B, 3D and 3E). These distortions of shape at the apical surface of hair cells were matched by complementary mis-shaping in the heads of the supporting cells that surround each hair cell so that they completely filled the spaces between hair cells to maintain a continuous, unbreached epithelial surface (Figs 1B, 3B and 3C, S1 Fig) but on occasions there was apparent direct contact between two hair cells at the surface of the epithelium (Fig 3F). The apical surface area of OHCs in the affected animals was slightly, but significantly, smaller than that of OHCs in normal animals (14.4±2.9 vs 16.6±2.8; p<0.001) (Fig 3I).

There were significantly fewer stereocilia in each OHC hair bundle in the affected animals than in those with normal bundles (61.2±10.1 [n = 245 bundles] vs 77.9 ±7 [n = 273 bundles] p<0.001) pooled across the entire length of the organ of Corti) (Fig 3I). There was a greater reduction in stereocilia numbers on OHCs of the basal coil in comparison with the apical coil because in normal animals there were more stereocilia on basal coil OHC than at the apex (86.3±9.1 at base, 73.4±9 at apex in normal animals; 61.2±9.4 and 61.3±11.1 respectively in the affected animals). Stereocilia were also shorter than normal (Fig 3C and 3I; S3 Fig) (0.9±0.12μm [n = 139] vs 1.2±0.18μm [n = 135] in the base; 1.2±0.2μm [n = 109] vs 1.8±0.5μm [n = 38] in the apical coil), and there was variability in the height of the outermost stereocilia in the bundle (lateral side) in comparison with normal bundles where all the outermost (longest) stereocilia were of the same height (S3 Fig). There was no obvious effect on the width of stereocilia (0.23±0.04μm [n = 81] in affected vs 0.2±0.03μm [n = 153] in normal animals) (Fig 3I). Although shorter than normal, the tallest stereocilia still reached, and were embedded in, the underside of the tectorial membrane, where the impressions of the embedded stereocilia reflected the variabilities of bundle shape and stereociliary height. In normal animals the underside of the tectorial membrane exhibited the single row of impressions of the longest stereocilia of each individual bundle organised along a distinct “W” shape (Fig 3G). In the affected animals (Fig 3H), there were fewer impressions for each individual hair bundle, the impressions formed a variety of shapes that matched those of the hair bundles and often there were two closely parallel rows of impressions from an individual bundle, rather than the normal single row, indicating a minimal height gradient between the rows of the longest stereocilia in an individual bundle.

#### Abnormalities of IHC bundles were different from those of OHC

**Fig 4:**
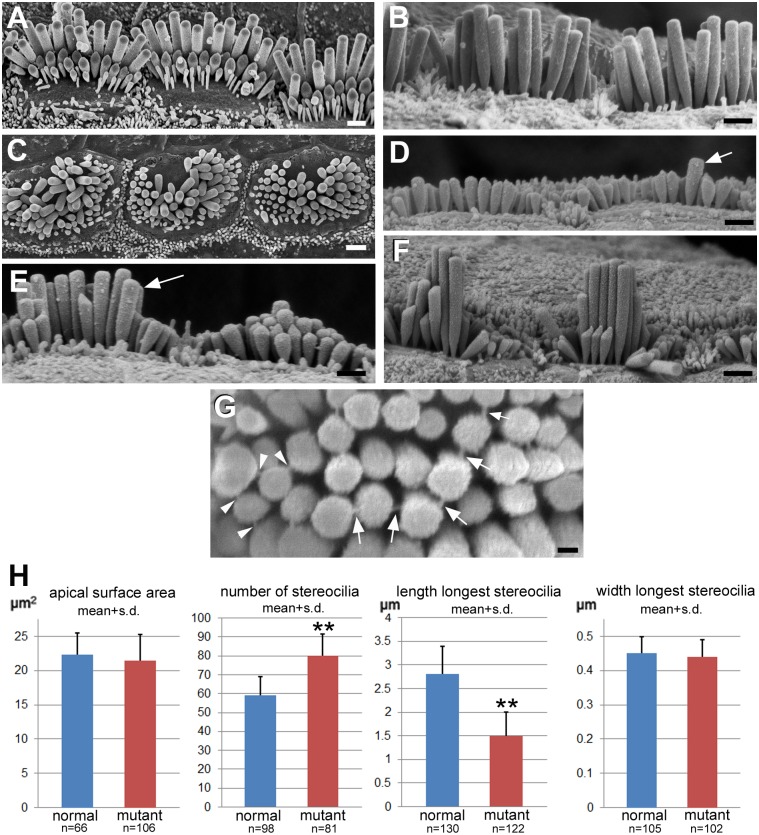
Inner hair cells. **A, B Normal animal at P17. C-G. Affected animals. C. (P25).** Apical surface of the cell and bundle in affected animals is more rounded than normal (A). The shape of the bundle is variable. There are stereocilia of different lengths but the longest ones are often in the centre of the bundle and there are no regular rows so there is no definable polarity as in the normal bundles. **D: Basal; E: Middle; F Apical coils (P17).** There is considerable variability in the heights of stereocilia on adjacent cells and height differentials in an individual bundle are often from the periphery towards the centre (E). Stereocilia in the affected animals are much shorter than those in equivalent regions in normal animals. Panel B shows IHC stereocilia in basal coil of normal littermate at the same magnification as the basal coil in the affected animal in panel D. There is, however an increase in height of the longest stereocilia along the organ of Corti from base to middle to apex (D,E,F, from same affected animal at same magnification). Arrows in panels D and E indicate examples of clearly longer, protruding stereocilia in bundles which were the only ones measured in affected animals for data used to estimate stereociliary lengths of stereocilia. **G. (P17).** Apparent tip-links (arrows) running from the top of a shorter stereocilium to the side of an adjacent longer one. Arrowheads indicate where one shorter stereocilium appears to form links with two longer neighbours. Note the variable heights within the cluster of longer stereocilia. Scale bars: A-F 1μm; G 0.25μm. **H.** Histograms displaying means of apical surface area, number of stereocilia and length and width of longest stereocilia on IHCs in normal and affected animals. Each mean was derived from pooled measurements/counts at basal, middle and apical coils in individual cochleae from at least 3 different animals aged P17-P22. N = number of cells assessed in each group. In affected animals, measurements of longest stereocilia were restricted only to those bundles where there were clearly longer stereocilia, protruding above their neighbours as arrowed in panels D and E. The result therefore does not show the true variability in lengths of stereocilia in the hair bundles of IHC in affected animals.

The normal IHC bundles in unaffected animals (Fig 4A and 4B) were elongated in the longitudinal direction along the organ of Corti, matching an elongated cell surface. The stereocilia formed a polarised, morphologically asymmetric bundle in rows of ascending height, and those on the inner side were usually quite thin, while those that formed the outermost longest row were much thicker (Fig 4B). In contrast, the hair bundles of IHC in the affected animals (Fig 4C) were rounded and the apical surface itself was more rounded than normal, but while the apical surface area was not significantly different from that of normal IHC (Fig 4H), the ratio of the greatest width of the hair cell surface in the longitudinal direction along the organ of Corti versus the width of that surface in the radial (lateral-medial direction) was significantly smaller (i.e. closer to circular where the ratio would be expected to be 1) in the affected animals than in unaffected, normal ones (1.5±0.2 [n = 87] vs 1.65±0.34 [n = 66] p = 0.001). Many of the bundles covered almost the whole of the surface but in others there was an area free of stereocilia usually located on the lateral side (towards the pillar cells) but this varied from cell to cell. There was a significantly greater number of stereocilia constituting an individual anomalous IHC hair bundle than in a normal one (80.2±11 [n = 82 bundles] in affected vs 59.3±9.7 [n = 98] in unaffected animals; p<0.001) (Fig 4H). In the bundles of some IHC, stereocilia were short with a semblance of height gradients while in others some stereocilia were significantly longer than others in the bundle, but the longest stereocilia were usually in the centre of the bundle (Fig 4C and 4E). There was thus no “polarity” of individual bundles in the sense of a defined asymmetry in a particular direction, and thus no consistent orientation of the bundles. The number of obviously longer stereocilia varied even between adjacent hair cells (Fig 4D and 4E). Stereocilia were significantly shorter than normal (compare normal basal coil IHC in Fig 4B with affected basal coil in Fig 4D), 1.5 ± 0.5 vs 2.84 ± 0.63 p<0.001 (for the entire length of the organ of Corti) (Fig 4H) although there was an increase in height of the longest stereocilia in the base to apical direction along the organ of Corti (Fig 4D, 4E and 4F). Nevertheless the longest stereocilia of IHC in affected cochleae at any location along the organ of Corti were always shorter than those at the equivalent position in normal animals (base: 1.5±0.5μm [n = 43] in affected vs 2.5±0.5μm [n = 70] in unaffected animals; apical: 2.7±0.6μm [n = 59] vs 3.5±0.3μm [n = 40]). Where there was obvious height differential, tip-links (which are thought to gate the transduction channel) were apparent between the tip of a shorter stereocilium and the shaft of an adjacent longer stereocilium, but in some cases a single shorter stereocilium appeared to form tip-link-like structures with more than one longer neighbour (Fig 4G). As with the OHC, the width of the longest stereocilia on IHC in the affected animals was no different from that of the longest stereocilia in normal bundles (mean, 0.44μm), so that the differential width difference between outer and inner hair cell stereocilia that is a feature of the normal organ of Corti was preserved.

#### Abnormalities were present in the bundles of immature cochlear hair cells

**Fig 5:**
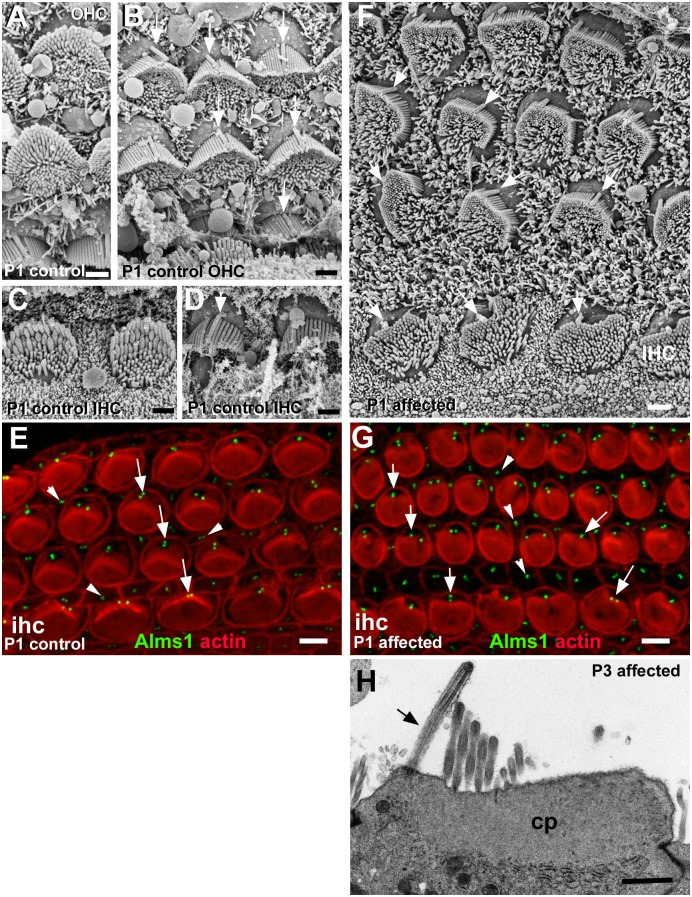
Morphology, polarity and orientation of hair bundles in early postnatal organ of Corti. **A-E: Normal at P1. A, B:** Outer hair cells**; C, D:** Inner hair cells. Hair bundles are polarised and uniformly oriented. **A**, **C:** Apical surface projections densely cover almost the entire rounded apical surfaces of each hair cell. Longer stereocilia in several rows toward the lateral side of the cell surface with a distinct height gradient form a curve with numerous shorter “microvilli” clustered inside. **B, D** Kinocilia (arrowed) are positioned behind the row of longest stereocilia, almost precisely at the apex of curve formed by the stereociliary bundle such that the positions of the kinocilia are aligned with hair bundle orientation. **E**. Whole-mount of a normal mouse organ of Corti. An anti-Alms1 antibody labelled basal bodies (mother and daughter centrioles) of hair cells (arrows) and supporting cells (arrowheads). The basal bodies are aligned in a regular arrangement at the lateral pole of the hair cells. **F, G: Affected at P1; H at P3. F.** Organ of Corti of an affected animal at P1. Bundles of outer hair cells are all variously misshapen. Several rows of stereocilia of increasing height around the outer side of distorted hemispheres enclose shorter microvilli covering the entire apical surface of the cell on the inner side. Individual bundles thus show polarity, but the longest stereocilia appear shorter than their counterparts in normal bundles. Stereociliary bundles broadly oriented towards the lateral side of the epithelium but are not precisely oriented. Kinocilia (arrowed) are located behind the longest stereocilia in a region of smooth apical surface membrane and are more regularly arranged than the stereociliary bundles suggesting a mis-alignment between kinociliary position and stereociliary bundle orientation. Bundles of inner hair cells (IHC) are more rounded and height gradients are less pronounced. **G.** Whole mount of an affected animal, littermate of the normal in panel E. The Alms1 antibody also stained basal bodies in hair cells (arrows) and supporting cells (arrowheads), which are in a comparable arrangement to controls. **H.** Thin section of apical end of an OHC from an affected animal at P3 shows the kinocilium (arrowed) at one edge of the cell arising from a location in a region free of the cuticular plate (cp) which underlies the stereocilia. The apical surface membrane to the outer side of the kinocilium is smooth. Scale bars; A,B,C,D,F&H 1μm; E,G 5μm.

**Fig 6:**
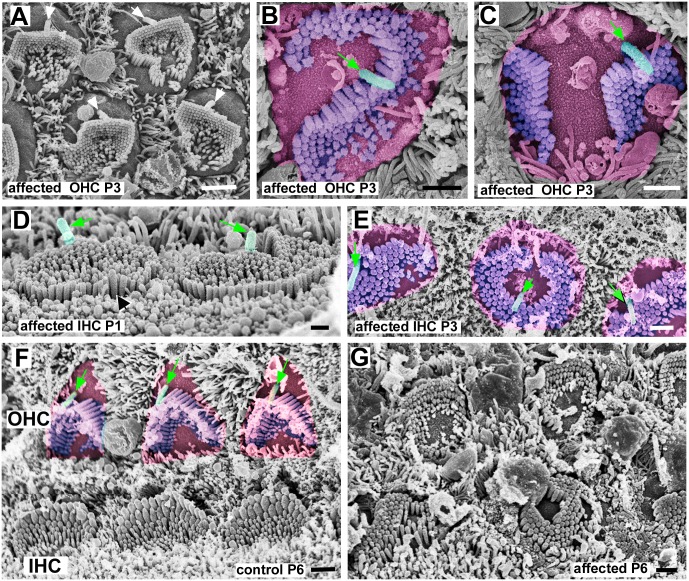
Progression of maturation of hair bundles in early postnatal ages. **A-C: Affected OHC at P3. A, Apical coil.** Bundles, more angular in contour than rounded, are completely surrounded by smooth apical surface membrane confining them to the centre of the apical surface., The apical surface of the cell is rounded. The bundles have adopted a variety of shapes and some show indications of dividing into separate clusters. Kinocilia are located behind the longest stereocilia at the lateral side and the arrangement of kinocilia on different hair cells is quite regular, more so than the orientation of the stereociliary bundles. **B & C, Basal coil.** Apical surface of the cell false coloured in pink, the sterociliary bundle in purple and the kinocilium in green (and arrowed). In line with the normal progression of maturation of hair cells from basal to apical coils, in affected animals the bundles in the basal coil appear more mature than those in the apical coil: there has been reduction in the number of stereocilia and the shape of the cell surface is more closely aligned with that of the stereociliary bundle. B. Stereocilia in rows of ascending height. The stereocilia curve around the kinocilium, which is located almost in the centre of the cell surface. C. Stereocilia in rows of ascending height in each of the two separated stereociliary bundles. The kinocilium is located behind the longest stereocilia of one bundle and located towards the lateral edge of the cell surface but to one side of the lateral-medial mid-line. **D&E: IHC in affected animals. D: P1.** Basal coil. Stereocilia are almost all of the same height although some slightly longer ones form the perimeter of the rounded bundle, and longest stereocilia (black arrow) sometimes on the side opposite to the kinocilium (false coloured green and arrowed). Kinocilia on the lateral side of the bundle. **E. P3.** Basal coil. Apical surfaces of hair cells (false coloured pink) adopting shapes resembling contours of their stereociliary bundles (purple) which vary in shape. Position of kinocilium (green and arrowed) varies; in some towards lateral edge of the cell surface and “behind” stereociliary bundle; in others in the centre of the cell surface surrounded by stereocilia. **F, Normal; G, affected: OHC and IHC at P6. F**. In outer hair cells (OHC) of a normal animal, most of the supernumerary microvilli to the medial side of the bundle have been lost to create a region of “smooth” apical surface membrane (false coloured pink) on that side. The stereociliary bundle (blue) has the mature shape; the bundle shapes are uniform across all OHC; and there is uniform bundle orientation, with the apex of the “M”-shape directed laterally. The shape of the apical surface of the cell (pink) conforms approximately to that of the hair bundle and a large, smooth area of apical membrane has been created on the lateral side of (behind) the bundle. Kinocilia (green and arrowed) are positioned closely behind the stereociliary bundle at its vertex and are regularly positioned in alignment with stereociliaru bundle orientation. Bundles of inner hair cells (IHC) are uniformly elongated in the longitudinal direction along the organ of Corti with a clear gradient in height of stereocilia towards the lateral side. **G.** In the affected littermate OHC bundles show a variety of orientations and shapes and there is a height gradient across the bundle. IHC show an even greater variety of shapes and arrangement of stereocilia. There are minimal height gradients. In some hair cells the height gradient is towards one side; in others towards the centre surrounding a central bare area of apical surface membrane. Stereocilia appear shorter than their counterparts in normal IHC at this age. Scale bars: 1μm in all figures.

In normal animals, in the earliest postnatal period (P0-P1) the apical surfaces both of OHC and of IHC were covered in presumptive stereocilia, but the bundles were distinctly polarised and oriented (Fig 5A–5D). There was a clear height gradient in 4–7 parallel rows of closely clustered stereocilia that formed the outer border of the bundle in a U-shape (Fig 5A and 5C). More widely spaced, elongated microvilli covered almost the entire apical surface to the inner side. The orientations of such polarised bundles on adjacent cells, as defined by the direction of the “U”, were closely aligned (Fig 5B and 5D). The kinocilium was positioned behind the row of longest stereocilia (Fig 5B and 5D) close to the bundle and almost exactly at the centre of the curve that the rounded bundle formed on the most immature cells, with an area free of stereocilia to the outer side, while the bundle of stereocilia/microvilli extended almost to the inner (medial) edge of the cell covering the entire surface on that side of the bundle. As the OHC matured its bundle assumed the “M”-shape and the kinocilium was positioned almost precisely at the indent of the “M” (Fig 6F). The area of smooth apical surface behind the kinocilium enlarged while elimination of the excess microvilli on the inner side, apparently through their withdrawal into the cell, created a smooth apical surface to the inner side. In parallel, changes in the shape of the apical surface and of the bundle matched each other to produce the systematically changing angulation of the arms of the “M”-shape along the basal-apical axis of the organ of Corti. Likewise, on IHC as the number of presumptive stereocilia decreased, the cell surface and the bundle became elongated in the longitudinal direction (Fig 6F).

In the affected animals, the immature hair cells were covered in elongated microvilli in similar large numbers to those found in control animals (Fig 5F) (at P6: OHC in affected: 113.2±3.7 [n = 5] vs unaffected: 116.2±3.7 [n = 5]; IHC: 110.7±15 [n = 6] vs 93.8±3 [n = 5]). In both IHC and OHC, kinocilia were located eccentrically towards the lateral (outer) edge of the hair cell surface (Fig 5F) within an area free of microvilli, in an approximately similar position to that in normal littermates. Labelling of the basal bodies (with an antibody to Alms1, an intrinsic protein of the basal body [17]) showed the distribution and arrangement of basal bodies in hair cells across and along the organ of Corti were similar to, but somewhat less regular than in normal animals (Fig 5E and 5G). The kinocilium was located in a region of the apical surface that was smooth and devoid of stereocilia (Fig 5F). Thin sections showed that in hair cells in affected animals the kinocilium was positioned at the lateral edge of the hair cell surface in a region of the apical cytoplasm free of the cuticular plate, that by P3 was well–developed beneath the stereocilia (Fig 5H). Thus, if defined with respect only to the position of the kinocilia/basal bodies there was retention of polarisation and orientation at the apical surface of the cochlear hair cells.

With further maturation, in OHC the bundle of elongated microvilli/presumptive stereocilia that initially covered almost the entire rounded apical surface, became almost completely surrounded by a distinct “bare” area free of microvilli, often confining the bundle to the centre of the cell surface (Fig 6A). The bundle itself consisted of closely clustered stereocilia in 5–7 rows forming the perimeter of an irregular “U”-like shape, often more angular than rounded, on the side facing the kinocilium, with more widely separated elongated “minivilli” of similar length to each other filling the space within and closing the opening of the “U” (Figs 5F and 6A). The clustered stereocilia were in rows of increasing height, but the height gradient was much less pronounced than in normal bundles. They thus exhibited polarity. However, bundle orientation as defined by the direction of that polarity and the relative position of the opening of the “U”-shape, varied widely between cells and was dissociated from the position of the kinocilium (Figs 5F and 6A). Nevertheless, although some bundles were almost circular and the shape of most of the others was irregular, the orientation of many bundles could be defined as within a 180° arc towards the lateral side; few bundles were obviously completely inverted with the longest stereocilia facing the medial side. From the earliest postnatal period, the stereociliary bundles showed most of the organisational disruptions that were evident in the mature organ of Corti, becoming pronounced by P3 (Fig 6A–6C). They exhibited a variety of morphologies: rounded, elongated, S-like shapes and divided into separated clusters of stereocilia (Fig 6A–6C). With progression of maturation, as the number of apical projections reduced, the areas devoid of stereocilia at the apical surface enlarged and the apical surface shape changed, bundles with a serpentine-like form often curved around the kinocilium which then appeared to be located rather more centrally at the cell surface than towards the lateral edge (Fig 6B). In divided bundles the stereocilia in each cluster were arranged in rows of ascending height with only a single kinocilium positioned behind the longest stereocilia of one cluster, but whilst the other stereociliary unit was equally “polarised” it was completely separated from the kinocilium (Fig 6C). There was thus a dissociation of bundle orientation and the position of the kinocilium.

In IHC, at P1-P3, the “bare” region at the apical surface was quite small and the rest of the surface was entirely covered with presumptive stereocilia (Figs 5F and 6D) so that the bundle of projections was essentially oval although there was considerable variability between cells in the shape of the bundle, some with stereocilia in a ring around a central bare area (Fig 6E). Stereocilia in several rows towards the site of the kinocilium were closely clustered while those further away were more widely separated (Fig 5F). The stereocilia were shorter than those of normal littermates. In some bundles there was a slight height gradient essentially in the direction of the kinocilium, indicating polarisation, but in others longer stereocilia were evident on the side opposite to the kinocilium or all around the periphery of circular bundles, and in still others all stereocilia were of almost equal height (Fig 6D). As maturation proceeded the apical surface of the cell and the bundle became more irregular in shape, with significant cell to cell variability in the morphology of the bundle (Fig 6E and 6G). The position of the kinocilium was also quite variable. Sometimes it was located towards the medial side, (i.e. the opposite to that which is normal and from where it was generally located at earlier times) or almost at the cell centre surrounded by stereocilia (Fig 6E). While often the kinocilium was within or associated with an area free of microvilli, small bare areas also developed at variable sites around the stereociliary bundle (Fig 6E). In comparison with the IHC bundles of normal litter mates, stereocilia were much shorter (Fig 6F and 6G) with only a minimal height gradient (best seen by stereoimaging, S4 Fig), if any, across the bundle and bundle morphology varied from elongated with a minimal height gradient in one direction (Fig 6E and 6G, S4 Fig) but not oriented with respect to bundles on adjacent cells; to rounded with slightly longer stereocilia in the centre sometimes clustered around the kinocilium (Fig 6E and 6G; S4 Fig); to irregular ill-definable shapes (Fig 6E and 6G). Thus, both polarisation of the stereociliary bundles, and consequently their orientation, were disrupted. However, cross-linking between stereocilia, similar to that seen in normal immature bundles and including apparent tip links was evident (S4 Fig).

### Hair bundles in utricular and saccular maculae

**Fig 7:**
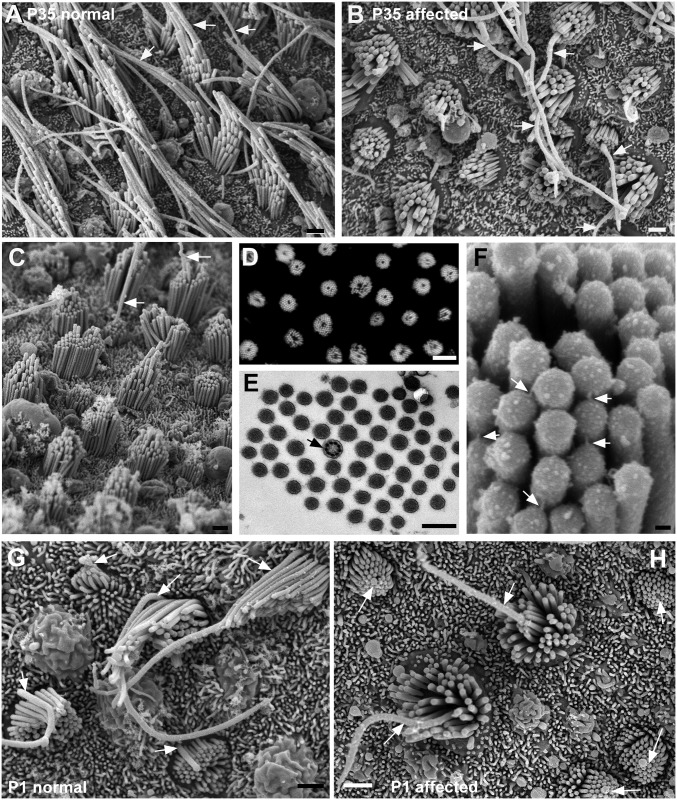
Utricular macula. **A. Normal.** Aged P35. Stereociliary bundles are elongated in shape. Well-defined gradients in the height of stereocilia across the bundle. The longest stereocilia adjacent to the kinocilium (arrowed) are very long. **B-F Affected animals. B.** P35 littermate of the normal animal shown in A. Bundles and apical surface rounded. Stereocilia appear shorter than normal. Long kinocilia (arrowed) emerge from the centre of some bundles, but in other bundles the kinocilium does not rise above the stereocilia. **C.** P42. A few bundles show distinct height gradients from periphery to centre of the bundle, but in the majority the height gradients are not marked or stereocilia are of similar height, Arrows indicate kinocilium emerging from the centre of stsreociliary bundles. **D**. Phalloidin labelling of actin in stereocilia in a whole mount preparation, shows every bundle in the field is affected, each bundle is round with the stereocilia surrounding a central unlabelled “hole”. **E.** Thin section across a hair bundle. Stereocilia surround a centrally located kinocilium (arrowed). **F.** Tip-link like structures (arrows) from tip of shorter stereocilium to side of adjacent longer oriented from periphery to centre. **G-H Maturation of hair bundles**. **G. Normal at P1.** Progressive stages in the maturation of the hair bundles are evident. In all bundles from the most immature with the shortest stereocilia, kinoclia (arrowed) are positioned eccentrically behind the longest stsreocilia. **H. Affected animal at P1.** In all bundles, from the most immature, kinocilia (arrowed) are located in the centre of the cell and are completely encircled by stereocilia which cover the entire apical surface of the cell. Scale bars: A,B,C 1μm; D 10μm; E 0.5μm; F 100nm; G, H,I 1μm.

In the stereociliary bundles of hair cells of the utricular maculae in normal animals (Fig 7A) the longest stereocilia were very long—more than 5μm in those across the striola (the region where the line of reversal of hair bundle orientation occurs), those in extrastriolar regions reaching ca 10 μm—and the height gradients of the rows down to the shortest stereocilia were quite distinct. The kinocilium was located behind the longest stereocilia. The number of stereocilia on the utricular hair cells in the normal animals was ca. 50 (50.5±11.5sd (n = 23;) 8 animals), the large variation (37–76) a likely reflection of different populations of bundles associated with the two different hair cell types [34, 35] (but this was not analysed).

In inner ears with anomalous hair bundles in the cochlea, hair bundles in maculae were substantially different from those of normal littermates. As on affected cochlear hair cells, stereocilia were significantly shorter than normal (Fig 7B and 7C): at P35 no stereocilium exceeded 3.5μm in height. Affected bundles were rounded in shape (Fig 7B, 7C and 7D) and the stereocilia entirely encircled the kinocilium which, unlike the kinocilia of affected cochlear hair cells, was located almost centrally (Fig 7B, 7C and 7E). There was thus no bundle orientation. In some cells the kinocilium was very long (Fig 7B) but in many others it was shorter than the longest stereocilia, so that it did not emerge above the stereociliary bundle (Fig 7B and 7C). Some bundles showed a small gradient in the height of stereocilia from the periphery towards the centre, i.e. the longest stereocilia were those closest to the kinocilium (Fig 7B, 7C and 7F), and tip links were arranged along this gradient (Fig 7F). However, unlike the affected bundles on cochlear hair cells, the number of stereocilia comprising each affected macular hair bundle, was the same as normal (52.9±8.9; range 38–79, n = 25/6 animals).

In vestibular maculae, hair cells are generated into the early postnatal period such that at P1-P2 all stages in the maturation of hair bundles are exhibited. In the utricular maculae of normal unaffected animals at this age (Fig 7G, S4E Fig) in many of the most immature bundles, the kinocilium was located eccentrically and “behind” the bundle of short stereocilia which even at this stage showed distinct height gradients. A change in the shape of the hair cell surface and of the shape of the hair bundles from rounded to the more elongate contour of the mature bundle (Fig 7A) was apparent co-incident with relocation of the kinocilium. In the utricular maculae of affected animals of the same age (Fig 7H; S4C, S4D and S4F Fig) always the bundle occupied almost the entire apical surface of the cell with the kinocilium located centrally at the cell’s surface encircled by a cluster of stereocilia of almost equal height. The kinocilium never became re-located to an eccentric position behind a clearly polarised bundle as occurs during normal maturation of macular hair cells [7]. The difference between the normal and affected bundles was particularly clearly seen by stereoimaging (S4E and S4F Fig).

### Hair bundles in cristae

**Fig 8:**
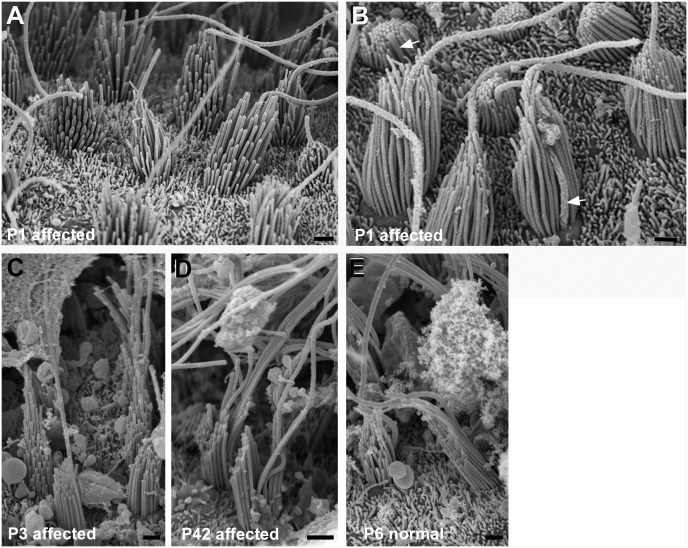
Cristae. Hair bundles in affected animals are polarised and uniformly oriented and appear similar to those in normal animals. **A,B: Affected animal P1.** Stereocilia increase in height in one direction across the bundle. All bundles are oriented the same way. B. In most bundles kinocilia are behind the longest row of stereocilia (arrows), even in those which are most immature, but in some other bundles the kinocilium is towards the “back” side of the bundle, but surrounded by stereocilia. **C. Affected animal P3.** Stereocilia (and kinocilia) have lengthened and there are clear height gradients in the bundle. **D. Affected animal P42.** Height gradients in stereocilia, longest stereocilia greatly lengthened. **E. Normal P6.** Scale bars: A-E 1μm.

The hair bundles of hair cells in the cristae appeared essentially unaffected in inner ears where bundles of cochlear and macular hair cells were abnormal. As early as P1 stereocilia were arranged in rows of ascending height and the direction in which the stereocilia increased in height, bundle orientation, was essentially the same for all hair cells (Fig 8A). The kinocilium appeared behind the longest streocilia in most bundles though sometimes could be seen to emerge from in front of the longest stereocilia (Fig 8B) suggesting a more central position and some variability in the relationship between the siting of the longest stereocilia and the position of the kinocilium. Nevertheless, the hair bundles of the cristae in affected animals showed both polarisation and orientation. By P3 (Fig 8C) the stereocilia (and the kinocilium) had grown rapidly to the same great lengths as in normal animals while retaining pronounced height gradients, so that at all ages examined up to 1 year, the hair bundles in the cristae of animals where cochlear and macular hair bundles were abnormal were indistinguishable from those in the cristae of normal littermates (Fig 8D and 8E).

### Abnormalities were confined to hair bundle organisation

**Fig 9:**
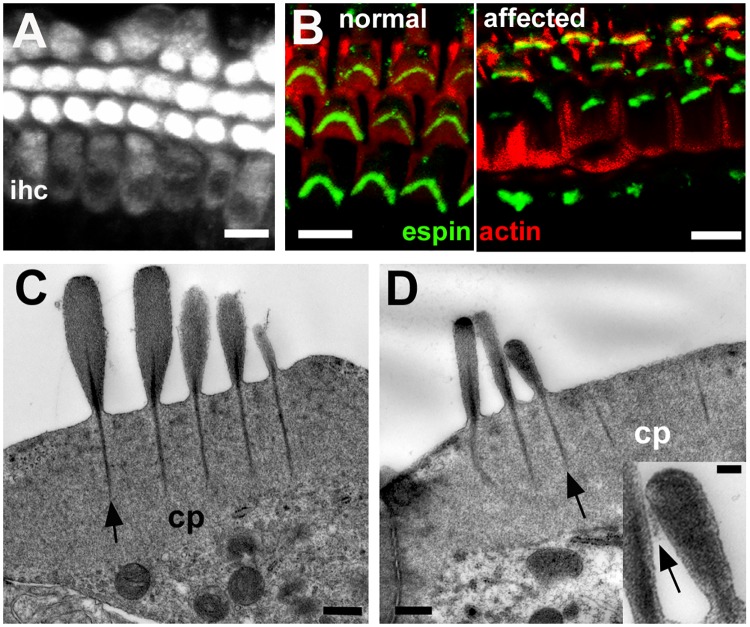
Defects are confined to hair bundle organisation. **A.** FM1-43 uptake in whole mount preparation of the organ of Corti of an affected animal at P10. After brief exposure to FM1-43, dye enters both outer hair cells and inner hair cells (ihc). Scale bar: 10μm. **B.** Immuno-labelling for espin (green) in whole mount preparation of the organ of Corti (actin labelled with rhodamine tagged phalloidin, red) of a normal and an affected animal at P22. Stereocilia in morphologically abnormal bundles label as intensely for espin as those in the normal littermate. Scale bar: 10μm. **C, D.** Thin sections of the apical end of inner (C) and outer (D) hair cells in affected animal at P30. Cuticular plates (cp) are well developed. Prominent stereociliary rootlets (arrows) descend from the shafts of the stereocilia into cuticular plates. Lateral cross-links between shafts of stereocilia arrowed in inset. The longest stereocilia in the bundle of the IHC (C) are quite short and there are two “long” stereocilia in adjacent rows of the same height. In the OHC (D) the two longer stereocilia in adjacent rows are of similar height while the shorter stereocilium appears unusually wide. In the inset the lateral crosslinking between adjacent stereocilia is indicated by the arrow. Scale bars: A,B 10μm; C, D 0.5μm, inset 0.1μm.

Brief exposure of the organ of Corti of affected animals to the styryl dye FM1-43, which is taken up into hair cells via the transduction channel at the tips of the stereocilia [36], resulted in fluorescent labelling of both IHC and OHC (Fig 9A) in an identical manner to normal hair cells [36], indicating that despite the bundle anomalies the stereocilia bore active transduction channels. Immunolabelling for the actin crosslinking protein espin, which when defective or absent causes anomalies of hair bundle morphology and stereociliary length [37], was present in the stereocilia of morphologically disrupted hair bundles (Fig 9B). Thin sections showed the filamentous actin in stereocilia was tightly packed as in normal stereocilia (Fig 9C and 9D) and “top connectors” [38] between the shafts of stereocilia at their apical ends were present (Fig 9D inset). These observations suggest that the defect did not affect the structure of individual stereocilia. Thin sections of the organ of Corti also suggested that morphological anomalies both of IHC and of OHC were confined to hair bundle organisation. The cuticular plates, upon which the stereocilia are mounted, were of the same size and apparently of the same density of actin cross-linking as in normal cochlear and macular hair cells (Fig 9C and 9D). Stereociliary rootlets running through the proximal end of each stereocilium into the cuticular plate were prominent (Fig 9C and 9D).

### Age-related changes

**Fig 10:**
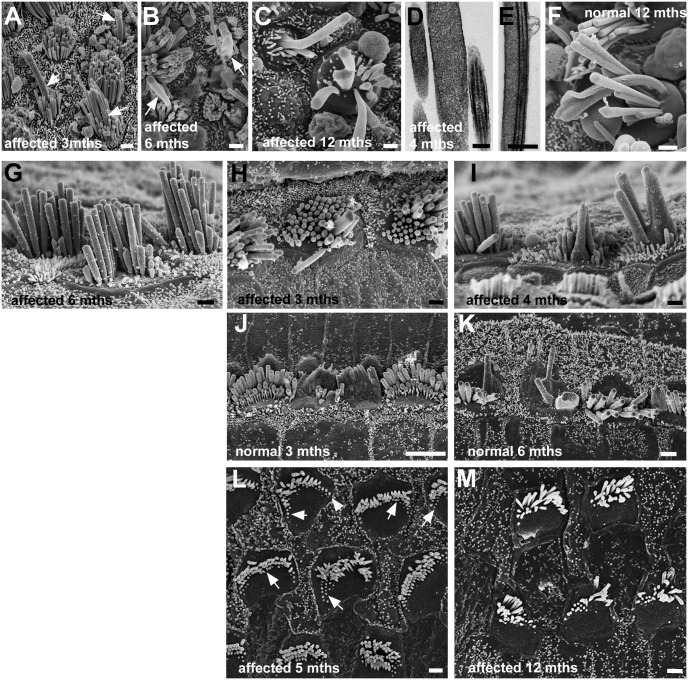
Ageing. **A-F: Utricles. A-E Affected animals: A: 3 months, B: 6 months, C: 1 year, D,E: 4 months. F: Normal animal at 1 year. A, B, C**. Long, wide projections initially from centre of bundles (arrows in A,B), suggesting fusion of stereocilium with kinocilium, get longer and wider and incorporate more stereocilia (C) with age. Thin sections, show wide “stereocilia” (C) indicating fusion, and fusion of kinocilium and stereocilia (D). Similar fusion and elongation of stereocilia occurs with ageing in normal animals (F). **G-K Cochlear inner hair cells. G-I: affected animals; J,K: normal animals. G.** In the apical coil of an affected animal at 6 months of age, stereocilia are longer (ca. 4–5μm) than their counterparts in the younger affected animals. **H, I:** Fusion and elongation of stereocilia in scattered hair cells in affected animals. H: 3 months, upper basal coil; I: 4 months, middle coil. **J, K:** Similar fusion and elongation occurs in hair bundles of scattered IHC in normal animals. J, 3 months, littermate of animal in H, upper basal coil; K upper basal coil; 6 months. **L,M. Cochlear outer hair cells in affected animal. L**: Apical coil at 5 months. Hair bundles show stumps of stereocilia particularly at outer edges (arrows) suggesting retraction into the cell. **M**: Apical coil at 1 year. There are fewer stereocilia in bundles and continuing apparent retraction is evident. Scale bars: A, B, C 1μm; D 0.2μm; E 0.5μm; F-I 1μm; J 5μm; K 2μm; L,M 1μm.

In the maculae of older animals with hair bundle anomalies, the length of stereocilia was greater than that in young mature animals; by P200 some stereocilia reached 4.5μm. Also, from ca. 3 months of age, an increasing number of hair bundles contained very long, thick structures indicating fusion and elongation of stereocilia and fusion of stereocilia with the kinocilium (Fig 10A–10E). Similar fused, elongated stereocilia were present in the maculae of older normal animals (Fig 10F) and have been described in other studies of age-related effects upon macular hair cells [39]. Likewise, stereocilia in the anomalous bundles of cochlear IHC, particularly those in the apical coils, appeared to increase in length with age; by 6 months the height of longer stereocilia in the bundles was ca. 5 μm (mean 4.8μm; n = 12 bundles) (Fig 10G). Elongated, fused stereocilia, similar to those in the maculae, also appeared in the anomalous hair bundles of cochlear IHC in older affected animals (Fig 10H and 10I) as well as in their normal litter mates where stereociliary fusion amongst IHC was evident from as early as 3 months of age (Fig 10J and 10K). However, neither increase in length nor fusion of stereocilia was obvious in the cochlear OHC in the older animals. With ageing, in animals with hair bundle anomalies there was an increasing number of OHC in which shortened and stump-like stereocilia were evident at the outer edges of the bundle (Fig 10L). This suggested a continuing retraction of stereocilia into the cell and a decrease in the number of stereocilia in the bundle. By one year of age (Fig 10M), the mean number of stereocilia in the bundles of apical coil OHC in affected animals was about half of that at P15-P30, 27.4±5.4 (n = 30 from 3 animals).

### Dysregulation of apical surface asymmetry signalling in affected hair cells

Since polarity and bundle orientation were disrupted in the organs of Corti and vestibular maculae of affected animals, the presence and distribution of core PCP [16, 40] and intrinsic bundle polarity pathway proteins [23, 25] were investigated by immuno-labelling these tissues from early postnatal animals. In the organ of Corti of the normal animals, the core PCP protein Vangl2 was localised in an asymmetrical pattern at the junction between hair cells and supporting cells (S5 Fig), as described elsewhere [40]. Vangl2 immunofluorescence was also present in the affected animals (S5 Fig), where it localised in a comparable pattern to that in normal animals, suggesting core PCP signalling was largely unaffected.

**Fig 11:**
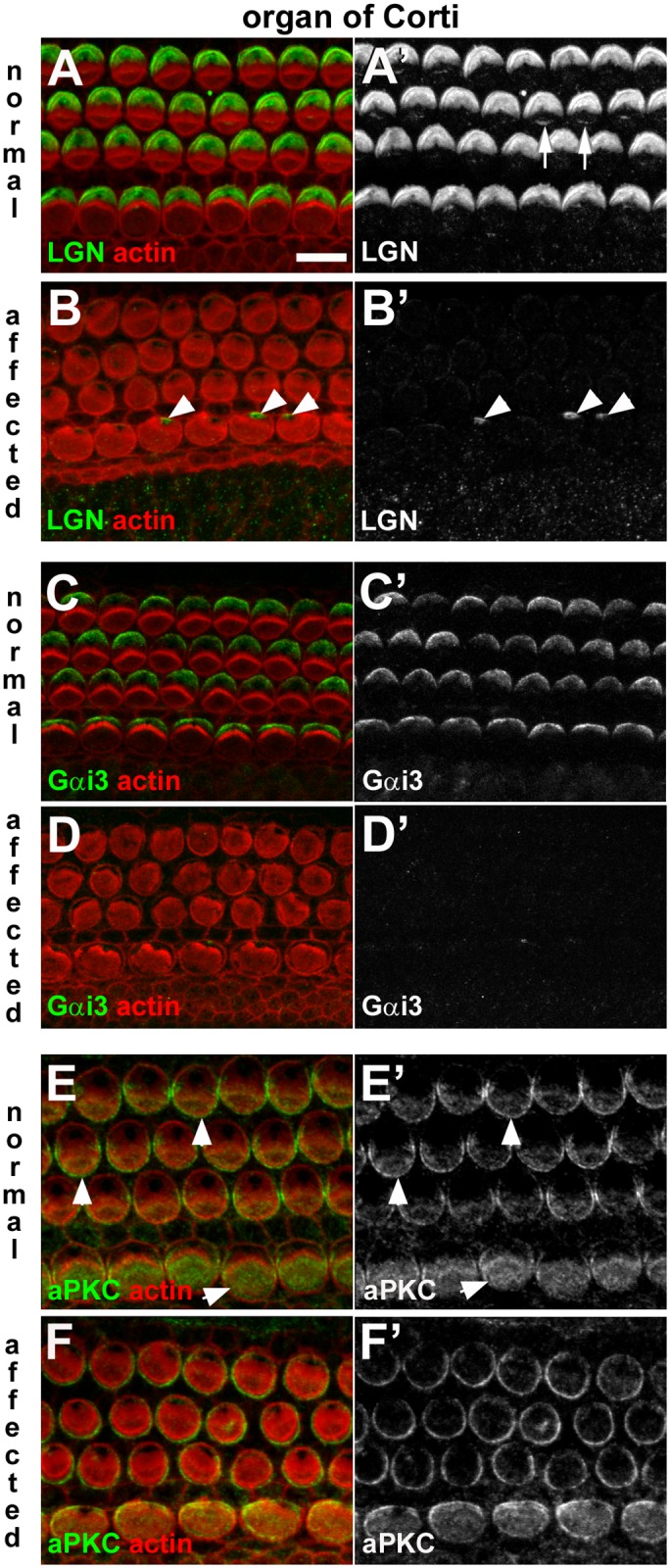
Dysregulation of apical surface asymmetry in cochlear hair cells of affected animals. **A-B: LGN in normal and affected animals.** In a whole-mount of P1 normal organ of Corti the anti-LGN antibody labelled the surface of OHC and IHC, specifically at the lateral pole (**A**, **A’**). Labelling at the tips of stereocilia also evident (arrows in **A’**). The asymmetric pattern of LGN distribution was not apparent in an affected littermate (**B, B’**). LGN immunofluorescence was detected in the peri-centriolar region of some IHC of affected animals (arrowheads in **B’**). **C-D: Gαi3 in normal and affected animals.** Gαi3 at the surface of hair cells in the normal organ of Corti (**C,C’**) showed the same asymmetric distribution as LGN. In affected animals labelling for Gαi3 is virtually undetectable in the organ of Corti (**D,D’**). **E-F: aPKC in normal and affected animals.** aPKC was detected at the surface of normal hair cells in the organ of Corti (**E,E’**), most strongly on the medial side (i.e. the side opposite to that of LGN and Gαi3 labelling) (arrowheads). In an affected littermate aPKC was detected in hair cells in the organ of Corti (**F,F’**), but the asymmetric distribution was not apparent. Scale bar in A (refers to all figures): 10 μm.

**Fig 12:**
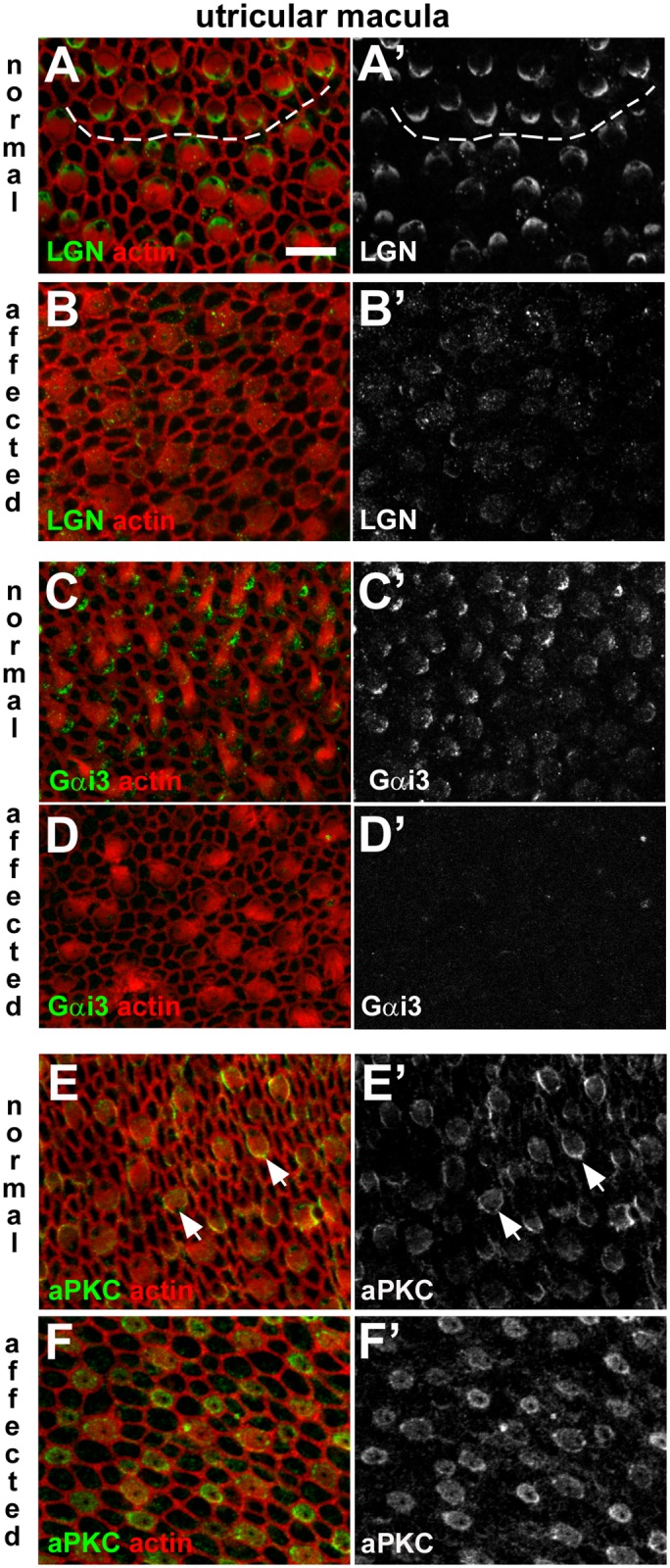
Dysregulation of apical surface asymmetry in utricular hair cells of affected animals. **A-B: LGN in normal and affected animals.** In a whole-mount of P1 normal utricular macula the anti-LGN antibody labelled the surface of hair cells, specifically at one side (**A**, **A’**). The micrograph details the striolar region in which hair cells of opposing orientation are apparent. The dashed line traces the line of polarity reversal. The asymmetric pattern of LGN immunofluorescence was not apparent in an affected littermate (**B, B’**). **C-D: Gαi3 in normal and affected animals.** Immuno-labelling for Gαi3 at the surface of hair cells in the normal utricular macula (**C,C’**) shows the same asymmetric distribution as LGN. In affected animals labelling for Gαi3 is virtually undetectable (**D,D’**). **E-F: aPKC in normal and affected animals.** aPKC was detected at the surface of hair cells in the normal utricular macula (**E,E’**), most strongly on the medial side (i.e. the side opposite to that of LGN and Gαi3 labelling; arrows). In an affected littermate aPKC was detected in hair cells (**F,F’**), but the asymmetric distribution was not apparent. Scale bar in A (refers to all figures): 10 μm.

**Fig 13:**
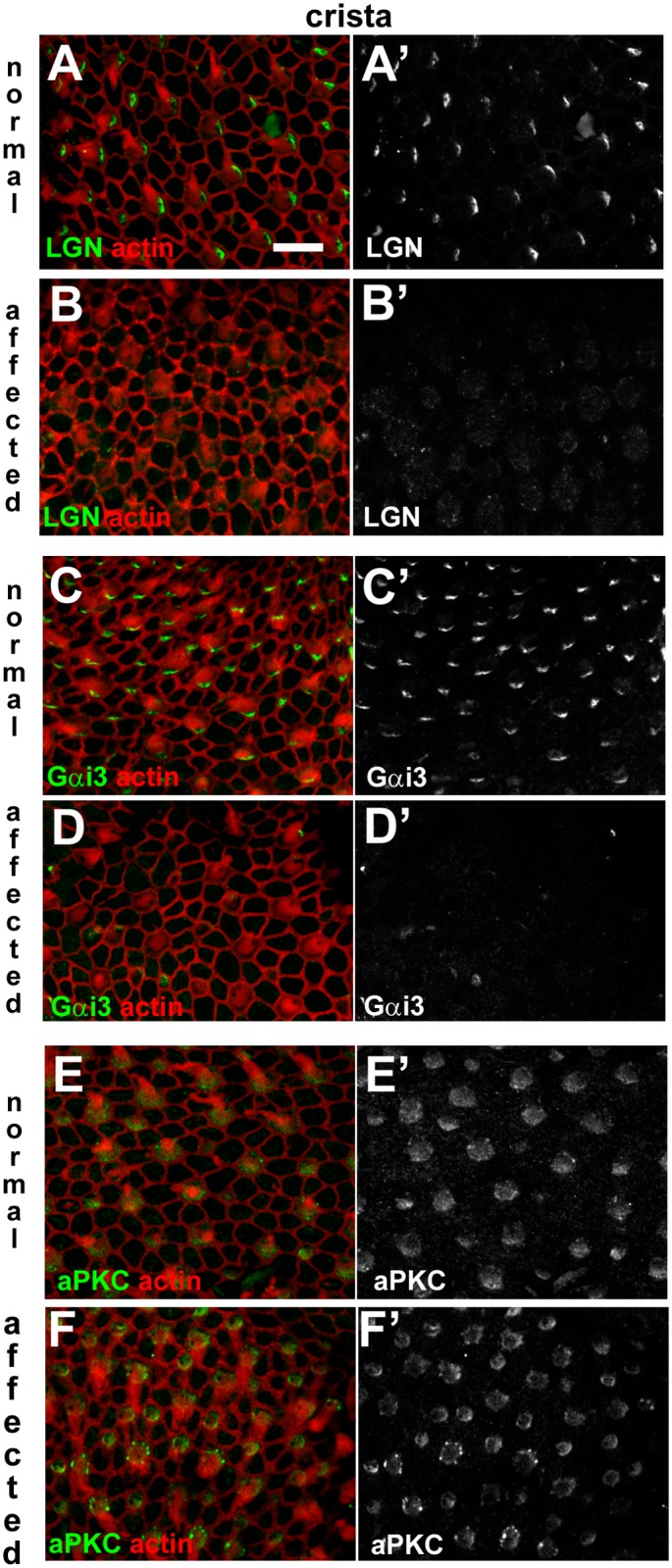
Dysregulation of apical surface asymmetry in crista ampullaris hair cells of affected animals. **A-B: LGN in normal and affected animals.** In a whole-mount of P1 normal crista ampullaris the anti-LGN antibody labelled the surface of hair cells, specifically at one side (**A**, **A’**). The asymmetric pattern of LGN immunofluorescence was not apparent in an affected littermate (**B, B’**). **C-D: Gαi3 in normal and affected animals.** Immuno-labelling for Gαi3 at the surface of hair cells in the normal crista ampullaris (**C,C’**) shows the same asymmetric distribution as LGN. In affected animals labelling for Gαi3 is virtually undetectable (**D,D’**). **E-F: aPKC in normal and affected animals.** aPKC was detected at the surface of normal hair cells in crista ampullaris (**E,E’**) most strongly on the medial side (i.e. the side opposite to that of LGN and Gαi3 labelling; arrows). In an affected littermate aPKC was detected in hair cells (**F,F’**), but the asymmetric distribution was not apparent. Scale bar in A (refers to all figures): 10 μm.

There were differences between control and affected animals, however, in the expression of proteins regulating the intrinsic hair bundle polarity pathway [23, 25] (Figs 11–13). In the normal organ of Corti LGN was localised asymmetrically, in a distinct lateral compartment at the apical surface of IHC and OHC (Fig 11A and 11A’), and it was also detectable at the tips of the longest stereocilia of OHC (Fig 11A’). In affected animals, immuno-labelling for LGN was almost entirely absent from the organ of Corti, save for a peri-centrosomal density in a few IHC (Fig 11B and 11B’). Immuno-labelling for Gαi3 localised to the same compartment as LGN in the normal organ of Corti (Fig 11C and 11C’), but was absent from the organ of Corti of affected animals (Fig 11D and 11D’). In normal animals, aPKC expression was restricted to a medial compartment at the apical surface of IHC and OHC (Fig 11E and 11E’). aPKC was present in the cochlear hair cells of affected animals, but it was evenly distributed around the entire circumference and across their apical surface (Fig 11F and 11F’).

LGN was detected in a lateral compartment on the apical surface of utricular hair cells of normal animals (Fig 12A and 12A’), but this asymmetric labelling was absent from hair cells of affected animals (Fig 12B and 12B’). Gαi3 was localised to a comparable lateral compartment in normal animals (Fig 12C and 12C’), but also was absent in the utricular hair cells of affected animals (Fig 12D and 12D’). aPKC expression was localised to one hemisphere of utricular hair cells of normal animals (Fig 12E and 12E’), and although aPKC was present in the utricular hair cells of affected animals it was evenly distributed around the entire circumference and across their apical surface (Fig 12F and 12F’). In cristae ampullares (Fig 13), the distribution of these proteins in normal animals, and their disruption in affected animals were comparable to those observed in utricular hair cells. Thus, disruption of SorCS2 is associated with loss or mis-expression of intrinsic polarity pathway proteins in the organ of Corti, vestibular maculae and cristae.

## Discussion

The likely proximal cause of the anomalous development of hair bundles described here is the disruption in *SorCS2*. Transcripts of *SorCS2* have been detected in cochlear tissue at E18 [41] and it is shown here that in normal animals not only is the gene expressed in the inner ear postnatally but also it is translated into protein in the organ of Corti, vestibular maculae and cristae from embryonic ages, when hair cells are first formed, through to the mature tissues. Its expression was severely reduced or absent from the inner ears of animals which exhibited anomalous bundles. The effect of the ~15 kb transgene insertion in intron 1 of *SorCS2* is difficult to predict but our qPCR data show that it severely reduces or abolishes expression of the major *SorCS2* transcript. This may be due to disruption of regulatory sequences required for normal expression or possibly effects on the efficiency of splicing. The result highlights the potential of unexpected, possibly deleterious consequences of uncontrolled, random insertions into the genome as an experimental approach.

### Roles of SorCS2

SorCS2 is widely expressed in the CNS [30, 42]. One role is as a co-receptor with p75NTR for pro-neurotrophins (NTs) mediating extracellular signals regulating actin dynamics to induce growth cone collapse and terminate neurite outgrowth [31, 33]. Genome-wide association studies have linked *SORCS2* as a risk factor for ADHD [43] which has been related to the role of the protein as a proNT receptor [33]. Likewise its proNT binding function may underlie an association of SorCS2 with epileptic seizures in mice [44]. However, members of the Vps10 family, to which SorCS2 belongs, also function in intracellular signalling and protein trafficking, sorting cargo from endosomes to the *trans*-Golgi-network or to the cell surface [45, 46]. Genetic variation in *SORCS2* has been associated with Alzheimer’s disease where the protein may affect the intracellular processing of amyloid precursor protein [46]. Nucleotide polymorphisms in *SORCS2* have also been associated with circulating levels of insulin-like growth factor binding protein 3 (IGFBP3) which regulates the level of IGF-1 in plasma [47]. Overexpression of IGFBP3 in mice leads to growth retardation [48]. The possibility that disruption of *SorCS2* affects the availability of IGF-1 thereby retarding growth could explain the present observation that the animals that showed hair bundle anomalies were invariably the lightest ones in litters.

In humans, *SORCS2* is located on the distal short arm of chromosome 4 within a gene-rich region subject to large chromosomal deletions that are associated with Wolf-Hirschhorn syndrome (WHS), a rare multisyndromic disorder [49]. The mouse homologues of the affected genes, including *SorCS2*, map to a syntenic region on mouse chromosome 5 [50, 51]. The nature and severity of the symptoms of WHS vary with the size of the deletion but they include seizures, skeletal defects that affect craniofacial development, growth retardation and hearing loss. While there is conductive hearing loss due to cranio-facial anomalies affecting the outer and middle ear, sensorineural hearing loss has also been recorded in ca. 15% of cases [52]. Recently this has been attributed to mutations in the gene encoding Wolf Hirschhorn Syndrome Candidate 1 (*WHSC1*) a histone methyl transferase that regulates gene expression [53]. In a mouse model, that mutation is associated with mis-orientation of hair bundles, but also, unlike the disruptions described here, in disturbance of the arrangement of OHC in the basal and middle cochlear turns. We suggest that in cases where its locus is included in the deletion, *SORCS2* is a second candidate gene that may underlie the sensorineural hearing loss in WHS through additional effects on hair bundle organisation.

### Interruption of intrinsic polarity signalling in hair cells

A consequence of disruption of *SorCS2* is loss of expression or mis-localisation of all the intrinsic polarity pathway proteins in hair cells, indicating SorCS2 likely plays a role in events upstream of their deployment. Whether it is acting as a receptor for some unidentified ligand, or whether it is involved in intracellular processing and/or trafficking of the proteins in hair cells is not known but our observations of cytoplasmic localisation of the protein at the time when hair bundles are forming may indicate a role in trafficking. Since the core PCP protein Vangl2 was asymmetrically localised in the normal pattern in both the cochlea and the vestibular maculae, it is likely that the defects in the intrinsic polarity pathway were the major factor contributing to the hair bundle anomalies. LGN and Gαi3, together with mInsc, are thought normally to form a complex [25]; loss of any one of them reduces, but does not ablate, expression of the others. Loss of expression of any one of them also produces effects that resemble those observed in the present study, but not as extensively since many OHC were only mildly or not affected and severe effects on IHC were not described [25]. The severity and extent of the hair bundle anomalies observed in the present study may, therefore, be because the expression or localisation of all the intrinsic polarity pathway proteins is affected simultaneously.

LGN, Gαi3 and mInsc are normally distributed together across the apical surface of the hair cell in the compartment at the “back” (kinocilial) side of the bundle, confining aPKC to the opposite hemisphere “in front” of the bundle [23, 25]. These complementary asymmetric patterns of distributions are thought to define the position of the bundle and to regulate its shape, as well as that of the apical surface of the hair cell, in particular that of OHC, through interactions with the acto-myosin cortical cytoskeleton beneath the apical surface plasma membrane [23, 25]. Polarised distributions of myosins and f-actin have been reported to guide co-ordinated reshaping of the contour of the apical surface and generation of the precise shape and orientation of the hair bundle that occur during the later stages of maturation of OHC [54]. The present work clearly shows a close relationship between the shape of the outline of the bundle and the contour of the apical surface in all affected hair cell types, most notably in OHC where the contour of the apical surface reflected the particular bundle shape even when it was quite tortuous or even split into separate clusters of stereocilia. Not only does this emphasise the close linkage between bundle shape and hair cell apical surface contour during the re-modelling. It also suggests that the loss of the intrinsic polarity proteins does not impair activity of the mechanisms underlying reshaping the apical surface of hair cells as they mature but leads to mis-regulation of the directions in which the forces underlying it are applied. This has implications for the development of the heads of the supporting cells that surround each hair cell. Normally, as the contour of the apical surface of the immature hair cell changes from round to the mature shape, the heads of the supporting cells undergo coincident remodelling to accommodate the shape changes in the hair cell. With loss or mis-expression of the intrinsic polarity proteins, the remodelling of the heads of the supporting cells accommodated the mis-shaping of the hair cell apex and maintained separation between adjacent hair cells, but occasionally it failed to do so resulting in apparent direct contact between neighbouring OHC. However, the normal maturation of supporting cells that results in systematic increases in the size of the heads of the pillar cells and of Deiters’ cells (the supporting cells in the region of the OHC) from base to apex of the cochlea [55] was unaffected.

Clearly, however, the hair bundles of cochlear IHC, cochlear OHC and macular hair cells are each affected differently by the same genetic defect and misexpression of intrinsic polarity proteins. Together with the observation that hair bundles in cristae were largely unaffected despite loss of these proteins. It would seem that there are differences between hair cell types in the way that the molecular machinery which generates a precisely organised hair bundle is deployed. In both outer and inner cochlear hair cells in affected animals at the earliest postnatal ages kinocilia were located eccentrically on the medial side of stereociliary bundles. Very early in the differentiation of cochlear hair cells, the kinocilium that is initially located towards the centre of the cell becomes positioned very close to one edge, before extensive expression of LGN/MInsc/Gαi3 [25] so is likely directed by the core PCP proteins [16] not those of the intrinsic polarity pathway. As differentiation proceeded and the shape of the cell surface changed, in IHC the kinocilium was initially close to the bundle and the edge of the cell, with only a small “bare area” free of microvilli around and behind it. Additionally, while there was some initial reduction in the number of presumptive stereocilia, no bare area was created on the opposite side of the bundle by retraction of stereocilia. Retraction appeared to be terminated early in IHC such that there was a significantly greater number of stereocilia in the bundle of adult animals than normal. The failure to generate the opposing smooth membrane areas either side of the bundle would be consistent with disruption of the projected role of the asymmetric distribution of intrinsic polarity pathway proteins in regulating delineation of bundle shape and position. In OHC, on the other hand, areas of smooth surface membrane were prominent in the region around the kinocilium, defining an “outer” side of the bundle, and on the opposite side through retraction of the supernumerary “minivilli” proceeding in a manner similar to normal as cell surface contour was re-shaped. In consequence, the hair bundle of the OHC retained a number of normal features: it became confined across the central region of the surface and to an almost normal number of rows of stereocilia; and it exhibited a “polarity” as defined by height differentials in one direction across the bundle (albeit streociliary lengths being significantly shorter than normal). Furthermore, in contrast to the situation in IHC, retraction of stereocilia not only proceeded but appeared to be excessive such that the total number of stereocilia on an OHC in adult animals was significantly less than normal, and retraction appeared to continue slowly throughout life, suggesting that the mechanism regulating retraction and defining the final number of stereocilia was not properly terminated. These observations suggest that in OHC there are elements additional to the intrinsic polarity pathway proteins that regulate hair bundle positioning and the number of rows of stereocilia.

In contrast to the situation in cochlear hair cells, in macula hair cells the kinocilium remained centrally at the surface surrounded by stereocilia. During normal maturation of stereociliary bundles in macular hair cells, the re-positioning of the kinocilium from the centre to one side of the bundle occurs as the apical surface area of the cell expands and the hair bundle re-shapes[7]. With the translocation of the stereocilia, the kinocilium becomes positioned behind the longest row. It is possible therefore, that normally rather than the kinocilium moving, it is fixed in position while, as the bundle reshapes and the apical surface expands, directed movements of the apical surface membrane around it leads to its eccentric position in relation to the bundle of stereocilia. A similar mechanism could account for the variable location of the kinocilium at the apical surface of IHC which occurred during apical surface re-modelling in affected animals. If the asymmetric distribution of intrinsic polarity proteins normally instructs directed movements of the apical surface membrane then it is possible that the retention of the kinocilium at the centre of the hair cell surface in macular hair cells in the affected animals is because of disruption of the apical surface membrane re-modelling as a consequence of mis-expression these proteins.

### Disruption of SorCS2 affects height of stereocilia

Although the shapes of bundles and the number of stereocilia of which they were comprised were affected, height differentials amongst stereocilia, though less marked than normal, were evident in individual anomalous bundles. In the bundles of macular hair cells, the longest stereocilia were those in the innermost ring encircling the central kinocilium. Such an organisation would be consistent with a hypothesis that the position of the kinocilium defines the location of the longest stereocilia in a bundle. In OHC differential heights were marked and the longest were in the row on that side of the bundle closer to the position of the kinocilium/basal body. In IHC, height differentials were initially less marked, and sometimes not obvious. With greater maturity, clearly longer stereocilia grew on many, but not all, bundles, with the longest stereocilia most commonly within the middle rather than the lateral (OHC-facing) side, as is normal. The re-location of the longest stereocilia to the middle of the bundle occurred as the shape of the apical surface changed from rounded to a more elongated, oval-like shape. Thus, the movements associated with re-modelling of the apical surface appeared to have led to re-distribution of stereocilia within a bundle, suggesting the position of stereocilia is not initially fixed. Since some of those longer, more centrally positioned stereocilia in a bundle increased markedly in length while others elongated little or not at all, it may imply that the longest stereocilia in the bundle are defined early and regardless of their position maintain the machinery to elongate differentially from their neighbours.

Another feature common to the abnormal hair bundles of inner and outer cochlear hair cells and hair cells of the maculae was a significant reduction in the length of stereocilia, without effects on width. Since the normal differential in width of stereocilia between IHC and OHC (and between vestibular hair cell types) was maintained in affected animals this implies that the number of actin filaments that form a stereocilium, and determine it width, is regulated through those genetic networks that define hair cell type separately from those that regulate bundle morphology. Stereocilia grow by the addition of monomers to the plus ends of actin filaments, which are located at the distal tips [3] and is thought to be regulated by a complex of proteins that includes myosin XVa [56]; whirlin, a scaffolding protein [57, 58]; and Esp8, which caps elongating actin filaments [59]. Severely shortened stereocilia result from the absence of any one of these proteins. Shortening of stereocilia may also occur with absence of actin cross-linking proteins fascin [60, 61] and most notably espin [37, 62]. However, labelling for espin was intense in stereocilia of the defective hair bundles of the organ of Corti and the packing of actin filaments within the stereocilia was unaffected suggesting that actin filaments are cross-linked normally. It has been reported (but not illustrated) that hair bundles in cristae, as well as those in maculae and the organ of Corti, show shortened stereocilia in the absence of myoXVa [56] but in the affected animals described here the stereocilia in the cristae grew to their normal great lengths. Thus, it may be that the molecular assembly that includes myoXVa is not directly affected by the mutation. In normal hair bundles, LGN and Gαi3 localise to the tips of the longest stereocilia [25, 63] and they may be additional players in the macromolecular assembly that regulates the length of stereocilia. Thus, the loss of expression of LGN and Gαi3 from inner ear sensory epithelia in the affected animals could be a contributory factor to the shortened stereocilia in the organ of Corti and maculae. However, if this is so then these proteins may not play a role in regulating stereociliary lengthening in cristae.

### Hair cells are not all created equally

Previous studies of hair bundle anomalies caused by defects in core PCP proteins, intrinsic polarity pathway proteins or in proteins involved in the formation of individual stereocilia, have not drawn particular attention to possible differences in the effects on the different sensory epithelia of the inner ear. Strikingly, although cristae normally appear to express SorCS2 and intrinsic polarity pathway proteins as in other sensory patches, the loss of these proteins did not have a significant effect on the morphology of their hair bundles when those in vestibular maculae and the organ of Corti were severely defective. Cristae are evolutionarily ancient in the vertebrate line—jawless fish possess only two cristae as well as a single common macula [64, 65]—and it could be argued they are functionally the simplest of the sensory epithelia in the inner ear since their role is solely vestibular whereas in bony fish and amphibia the saccular macula, and in some fish species the utricular macula as well, have a role in audition in addition to detecting position in space [66]. Furthermore, hair bundles in an individual crista are all oriented in the same direction and appear to be of essentially uniform morphology (except, in mammals at least, for differences in stereociliary width, associated with the two hair cell types, and length related to their location across the epithelium). In contrast bundles of macular hair cells show more than one orientation and often more than one distinct morphological type [67]. Thus, the machinery generating a bundle in the crista may be in its simplest form whereas the requirements for more precise bundle organisation with the acquisition of greater functional specialisation in maculae and auditory epithelia may have led to increasing/additional levels of regulation during hair bundle formation. It may be that cristae provide a better model than other sensory patches to explore the basic molecular machinery by which hair bundle polarity and orientation are established.

## Materials and methods

### Ethics statement

The work involving animals was performed in accordance with guidelines laid out by the British Home Office and with commitment to the application of the principle of replacement, reduction and refinement in the use of animals in research. In accordance with this commitment the number of animals used was the minimum needed to achieve the results sought; and the procedures used were refined as much as possible to minimise suffering of the animals used.

The work with animals formed part of a project that had been approved by the UCL Animal Welfare and Ethical Review Body (AWERB) prior to the award of a Project Licence from the British Home Office, No. PPL70/8144, issued in accordance with United Kingdom Animal (Scientific Procedures) Act of 1986 that gave approval of the work undertaken.

### Animals

The initial colony of mice consisted of *Tie2-cre* transgenic animals that were crossed with animals containing floxed *CNP* (c-type natriuretic peptide), both strains on a C57Bl/6 background. The colony included all possible homozygous and heterozygous genotypes of these animals as well as wild types. They were obtained from Prof Adrian Hobbs (Pharmacology Dept UCL, now at Queen Mary University of London) and were bred and maintained in the UCL Biological Services Unit. All procedures involving the use of animals were approved by the UCL Animal Ethics Committee and were performed under the terms of a project licence granted by the British Home Office. Screening of animals was performed by the “ear twitch response” to an 18.5kHz tone burst delivered from a “click box” [68] which initial ABR analyses showed was a reliable indicator of affected animals. Animals that showed no response were selected as “affected” animals and subsequent morphological analyses of their inner ears, or those of their offspring, were used to confirm phenotypes. In all further work, phenotypic assessment of one ear from each animal was used to identify affected animals. Early postnatal animals were from the litters of breeding pairs the genotypes of which had been determined from assessment of responses to click-box testing and the Mendelian ratios of the phenotypes in previous litters.

### Auditory Brainstem Responses (ABR)

ABRs were recorded blind to phenotype. Animals aged P17- P35 were anaesthetized and placed in a sound isolated chamber. Subdermal needle electrodes (Rochester Electro-Medical) were inserted at the vertex (active), mastoid (reference) and with the ground needle electrode in the hind leg. Recordings were obtained using TDT system3 equipment and software (Tucker-Davis Tech., Alachua FL). Responses to click stimuli and to tone pips at 8,12,24,32 and 40 kHz were recorded and threshold determined by the lowest level at which the ABR waveform could be recognised.

### Tissue preparation

The auditory bullae of mice of various ages from P0 to 1 year were isolated and the cochleae exposed. To provide access for fixative to the inner ear tissues, an opening was made at the apex of the cochlea, the vestibule was widely opened at the round and oval windows and small cuts were made through the lateral wall of the cochlea. Fixative was slowly injected into the inner ear via the openings at base and apex of the cochlea and then the opened bulla was immersed in fixative. Inner ears were also obtained from animals of embryonic ages E15-E18. Their heads were bisected longitudinally and placed in fixative. The fixative was either 2–4% paraformaldehyde in phosphate buffered saline (PBS) prior to immunohistochemical labelling, or 2.5% glutaraldehyde in 0.1M cacodylate buffer, pH7.3 with 3mM CaCl_2_ in preparation for electron microscopy. Fixation continued for a maximum of 2 hours at room temperature. The bullae from animals older than 10 days were decalcified in 4% EDTA in either PBS or cacodylate buffer, pH7.3, as appropriate, for no more than 48 hours, before further processing.

### Electron microscopy

For scanning electron microscopy (SEM) organs of Corti, utricular and saccular maculae and cristae were dissected from the bullae and postfixed in 1% OsO_4_ in cacodylate buffer. Samples were then processed through the thiocarbohydrazide-OsO_4_ repeated procedure [69] before dehydration in an ethanol series, critical point drying and mounting on SEM sample stubs with conductive silver paint. A thin (2-3nm) layer of Pt was applied by sputter coating before examination. For transmission EM (TEM) of thin sections in some cases tannic acid at 1% was added to the glutaraldehyde fixative. Bullae were trimmed to remove excess material from around the cochlea and vestibular system, which were partially opened, and the bullae were then processed intact without isolation of the inner ear tissues. The samples were post-fixed in 1% OsO_4_, partially dehydrated in an ethanol series to 70% ethanol, incubated overnight at 4°C in a saturated solution of uranyl acetate in 70% ethanol before completing dehydration and embedding in plastic. Thin sections of the entire cochlea were cut.

### Quantitative analysis

From SEM images, the number of stereocilia in individual IHC and OHC and the surface areas of these cells were obtained from organ of Corti samples tilted and rotated in the microscope to provide views from as close as possible 90° to the apical surface of the hair cells (i.e. from directly above) for surface area or additionally for counting stereocilia towards the inner aspect of the bundles, when all stereocilia in a bundle are visible. Images were collected at a nominal consistent magnification of 4000x, which provided for all three rows of OHC to be included in a single image. The height and width of IHC and OHC stereocilia were measured from images of samples tilted and rotated to view the stereocilia as closely as possible perpendicular to their long axes to reduce the effects of parallax on measurements. Images were obtained at a nominal consistent magnification of 10000x. Microscope magnification was calibrated with a cross-grating replica. To enable comparisons between animals in the location along the organ of Corti from which the quantitative data was obtained, the measured width of the inner pillar cell head between the IHC and first row of OHC, and that of the OHC region from the medial border of the first row of OHC to the lateral border of the 3^rd^ row OHC were used as surrogates for position; the width of the pillar cell heads and the width across the OHC region increase systematically from base to apex along the cochlea [55] and thus provide an indicator of relative position along the organ of Corti. Generally three images for each assessment at each location for each animal were obtained from 5 different litters. Counting and measurements were made from the micrographs with the aid of AnalySIS image analysis software. Statistical comparison was by t-test. Results are presented as mean ± standard deviation, and significance level was set at p<0.05.

### Immunohistochemistry

Organs of Corti, utricular maculae and cristae were dissected from the bullae, after decalcification when used, and prepared for whole mount examination. Entire opened bullae were prepared for vibratome sectioning and sections 150μm thick were cut. Whole mount samples or sections were permeabilised and blocked with 0.1% Triton X-100 and 10% normal goat serum in PBS for 30 minutes at room temperature, before overnight incubation at 4°C with primary antibodies diluted in 100 mM L-lysine in PBS solution. Following extensive washing in PBS, samples were incubated with appropriate Alexa-conjugated secondary antibodies (Invitrogen, Paisley, UK) diluted at 1:400 in 100 mM L-lysine in PBS solution for 1 hour at room temperature. Fluorescently-tagged phalloidin (Sigma) was added to the secondary antibody solution at 1 μg/ml. The samples were mounted using Vectashield (Vector Laboratories) containing DAPI to label nuclei. Samples were imaged using a laser scanning confocal microscope (LSM 510; Zeiss, Jena, Germany) or by wide-field fluorescence microscopy.

The primary antibodies used were: rabbit polyclonal anti-espin (1:100; kind gift of James Bartles, Northwestern University, Chicago, USA); rabbit polyclonal anti-Alms1 (1:100; gift from David Wilson, University of Southampton, UK); rabbit polyclonal anti-LGN (1:400; gift from Fumio Matsuzaki, RIKEN Center for Developmental Biology, Kobe, Japan); rabbit polyclonal anti-Gαi3 (1:400; Sigma Aldrich, Poole, UK; G4040); rabbit polyclonal anti-atypical Protein Kinase C (1:400; Santa Cruz Biotechnology Inc, Heidelberg, Germany; sc-216, C-20); rabbit polyclonal anti-SorCS2-CT (cytoplasmic tail) (1:1000; gift from Simon Glerup, Aarhus University, Denmark); sheep polyclonal anti-SorCS2-ED (extracellular domain) (1:50; R&D Systems, Abingdon, UK; AF4237); rabbit polyclonal anti-Vangl2 (1:500; gift from Mireille Montcouquiol, INSERM, Bordeaux, France).

### FM1-43 labelling

Segments of organ of Corti were isolated from the cochleae of animals aged P10 under Hepes buffered Hanks Balanced salt solution (HBHBSS). They were immersed in 3μM FM1-43 (ThermoFisher Scientific) for 20 secs, immediately transferred to HBHBSS and washed three times before examination by confocal microscopy.

### Whole genome sequencing

Tail snips from animals that showed the abnormal phenotype were assessed. Genomic DNA was extracted. This was fragmented to ~300bp and was processed for Illumina Next Gen DNA sequencing by standard library preparation protocols. The resulting library was DNA sequenced in one lane of an Illumina 2500 sequencer and yielded ~100 million paired end reads, each of 100 bases. The Fastq files from this were converted to a Fasta database. These were searched by BLAST for sequences homologous to the transgene (the Tie2 promoter, Cre cDNA, MT-1 polyA signal sequence, and Tie2 intron 1 enhancer). All of the resulting detected paired end sequence reads were extracted and further analyzed.

### RT-qPCR

Auditory bullae were obtained from mice aged P0-P4. One bulla from each mouse was removed to RNA-later (Qiagen) and stored at -80°C until analysis. The opposite ear from each of these animals was fixed in paraformaldehyde and processed to determine the inner ear phenotype. Nine heterozygote and 9 homozygote animals were included in gene expression analysis. The qPCR analysis was performed blind to the phenotype. RNA was extracted using an RNeasy kit (Qiagen), treated with RQ1 RNase-free DNase (Promega), and cDNA made using Omniscript reverse transcriptase (Qiagen) and using random primers (Promega). Relative levels of *SorCS2* gene expression were determined by RT-qPCR using Taqman^®^ gene expression assays from Thermo Fisher Scientific [assay ID: Mm00473051_m1 and Mm00473072_m1] to detect the 5’ and 3’ regions of the major predicted transcript respectively, and *Myo7a* (assay ID: Rn00596450_m1). Reactions were multiplexed with a primer-limited eukaryotic 18S rRNA endogenous control, performed in triplicate for each animal and amplified on a SDS7500 real-time PCR System (Thermo Fisher Scientific). Relative quantification studies were performed using SDS1.2.1 software using the 2^-ΔΔ*CT*^ method (Thermo Fisher Scientific).

## Supporting information

S1 Fig Organ of Corti in apical (A) and basal (B) cochlear coil of the same affected animal at P17. In the apical coil, while all OHC bundles are mis-shapen the effects appear less severe than in the basal coil. The widths across the inner pillar cell (ipc) and across the outer hair cell region (ohc) are much greater in the apical coil than at the base as in normal animals, suggesting maturation of the organ of Corti supporting cells that produces systematic changes in the dimensions of the organ of Corti related to tonotopicity is unimpaired in affected animals.
Scale bars: 10μm.
(TIF)

S2 Fig SorCS2 expression in sensory epithelia of the inner ear. **A-B.** Immunofluorescence using a rabbit antibody targeting the cytoplasmic tail of SorCS2 (SorCS2-CT). In the wild type organ of Corti at P1, the SorCS2-CT antibody localised to supporting cells surrounding the OHC, and to IHC and their supporting cells (**A,A’**). The confocal image was taken at the level of the hair cell nuclei (stained with DAPI, blue). This antibody localised primarily to hair cells in the utricular macula of the same animal (**B,B’**).
**C-F**. Immunofluorescence in cochlear vibratome sections of normal and abnormal animals at P1 using a sheep antibody targeting the extracellular domain of SorCS2 (SorCS2-ED). In the basal turn (**C,C’**) and apical turn (**D,D’**) of normal animals the SorCS2-ED antibody localised to hair cells and supporting cells. Nuclei were stained using DAPI (red). SorCS2-ED immunofluorescence was not detected in the basal turn (**E,E’**) or apical turn (**F,F’**) of affected animals.
**G.** SorCS2-ED immunofluorescence in a cochlear vibratome section of a P30 wild type mouse. The SorCS2-ED antibody localised primarily to supporting cells.
Scale bars: 10 μm.
(TIF)

S3 Fig Outer hair cells in upper basal coil of (A) normal and (B) affected littermates at P22. In normal animals the stereocilia that comprise the outermost (lateral side) row are all of almost the same height, whereas in the affected animal there is considerable variability in the length of these longest stereocilia.
Scale bars: 1μm.
(TIF)

S4 Fig **A,B.** Anaglyph stereoimages of IHC hair bundles of an affected animal at P6. **A**. Height gradient across the bundle from inner to outer side. **B**. Height gradient of stereocilia towards kinocilium located in the centre of the cell surface. Gradient is thus, from periphery to centre, the opposite to that in A. Various cross links between stereocilia are evident.
Scale bars: 1μm.
**C-F Hair bundle maturation in utricular maculae at P1**.
**C.** Thin section of hair cell in affected animal at P1. Kinocilium (arrowed) in centre of bundle **B.** Hair bundles in affected animal at P1. Entire, round apical surface of hair cell covered in stereociia of almost equal height with kinocilium (arrow) emerging from the centre. **C, D.** Anaglyph stereoimages. In the macula from a normal animal (C) all the kinocilia (arrows) are located eccentrically at one side of the bundle of stereocilia which show progressive stages of maturation on different hair cells. In the affected macula (D) in every bundle from, those with the shortest stereocilia to those with the longest, the kinocilium (arrowed) arises from the centre of the bundle at approximately the centre of the apical surface of the hair cell.
Scale bars: A,B 1μm; C,D 2μm.
(TIF)

S5 Fig Long-distance PCP signalling is preserved in affected mice. **A**, in a whole-mount of a normal (control) mouse organ of Corti an anti-Vangl2 antibody labelled apical junctions between supporting cells and hair cells (*arrows*). **B**, in an affected littermate the Vangl2 antibody stained comparable structures.
Scale bar: 10μm (refers to all panels).
(TIF)
