# Supplementary figures and images for: Disruption of SorCS2 reveals differences in the regulation of stereociliary bundle formation between hair cell types in the inner ear
Source: PLoS Genet. 2017 Mar 27;13(3):e1006692. doi: 10.1371/journal.pgen.1006692 (PMC5386298; doi:10.1371/journal.pgen.1006692)

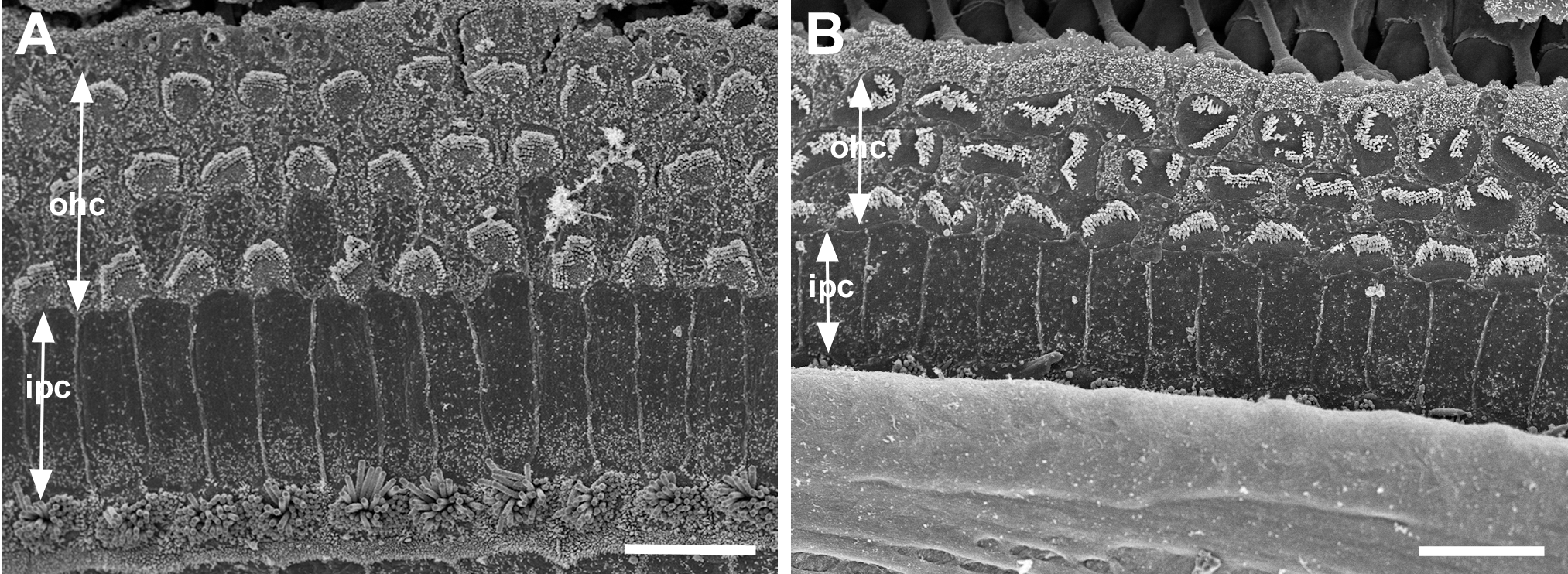

Supplement: S1 Fig — In the apical coil, while all OHC bundles are mis-shapen the effects appear less severe than in the basal coil. The widths across the inner pillar cell (ipc) and across the outer hair cell region (ohc) are much greater in the apical coil than at the base as in normal animals, suggesting maturation of the organ of Corti supporting cells that produces systematic changes in the dimensions of the organ of Corti related to tonotopicity is unimpaired in affected animals. Scale bars: 10μm. (TIF) [file pgen.1006692.s001.tif]

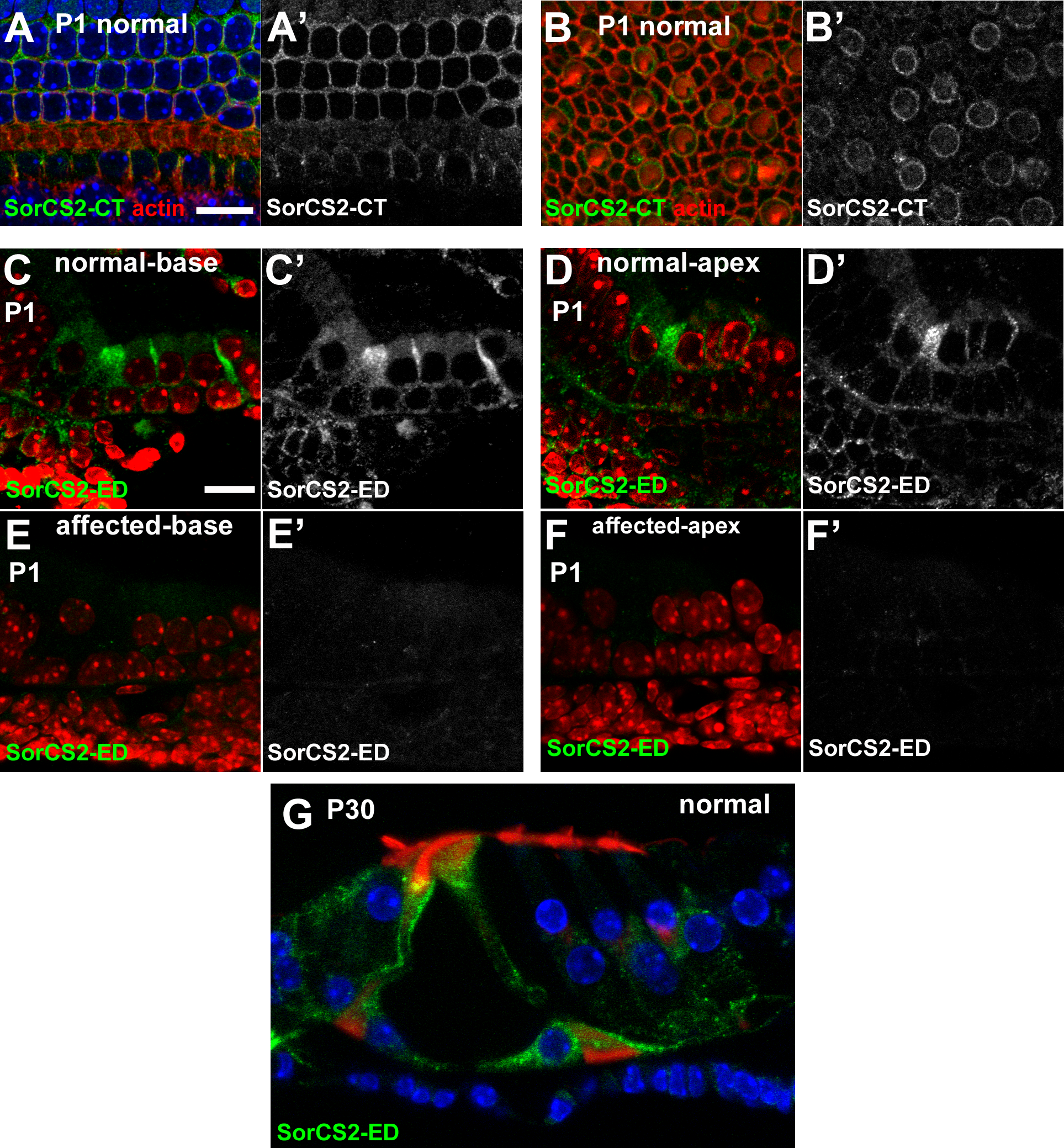

Supplement: S2 Fig — A-B. Immunofluorescence using a rabbit antibody targeting the cytoplasmic tail of SorCS2 (SorCS2-CT). In the wild type organ of Corti at P1, the SorCS2-CT antibody localised to supporting cells surrounding the OHC, and to IHC and their supporting cells (A,A’). The confocal image was taken at the level of the hair cell nuclei (stained with DAPI, blue). This antibody localised primarily to hair cells in the utricular macula of the same animal (B,B’). C-F. Immunofluorescence in cochlear vibratome sections of normal and abnormal animals at P1 using a sheep antibody targeting the extracellular domain of SorCS2 (SorCS2-ED). In the basal turn (C,C’) and apical turn (D,D’) of normal animals the SorCS2-ED antibody localised to hair cells and supporting cells. Nuclei were stained using DAPI (red). SorCS2-ED immunofluorescence was not detected in the basal turn (E,E’) or apical turn (F,F’) of affected animals. G. SorCS2-ED immunofluorescence in a cochlear vibratome section of a P30 wild type mouse. The SorCS2-ED antibody localised primarily to supporting cells. Scale bars: 10 μm. (TIF) [file pgen.1006692.s002.tif]

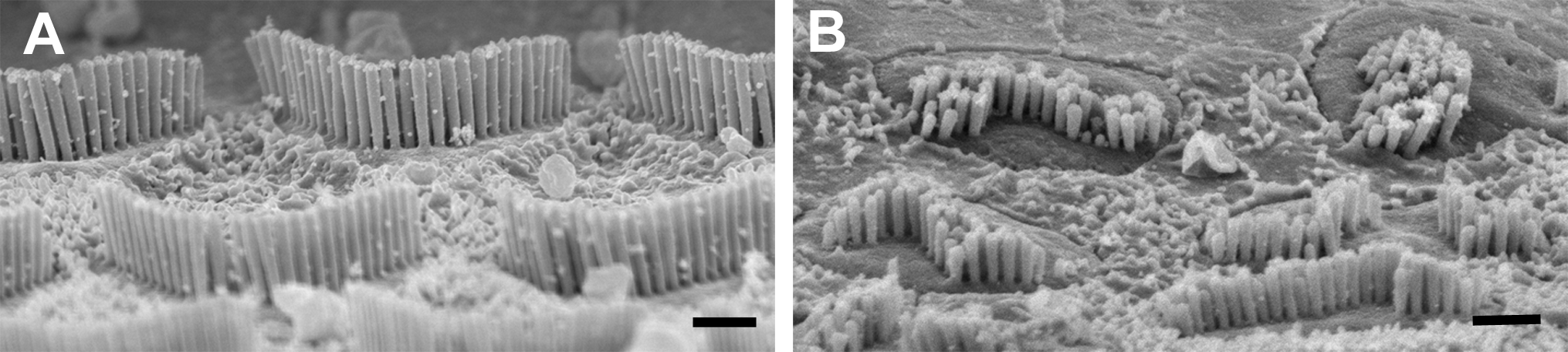

Supplement: S3 Fig — In normal animals the stereocilia that comprise the outermost (lateral side) row are all of almost the same height, whereas in the affected animal there is considerable variability in the length of these longest stereocilia. Scale bars: 1μm. (TIF) [file pgen.1006692.s003.tif]

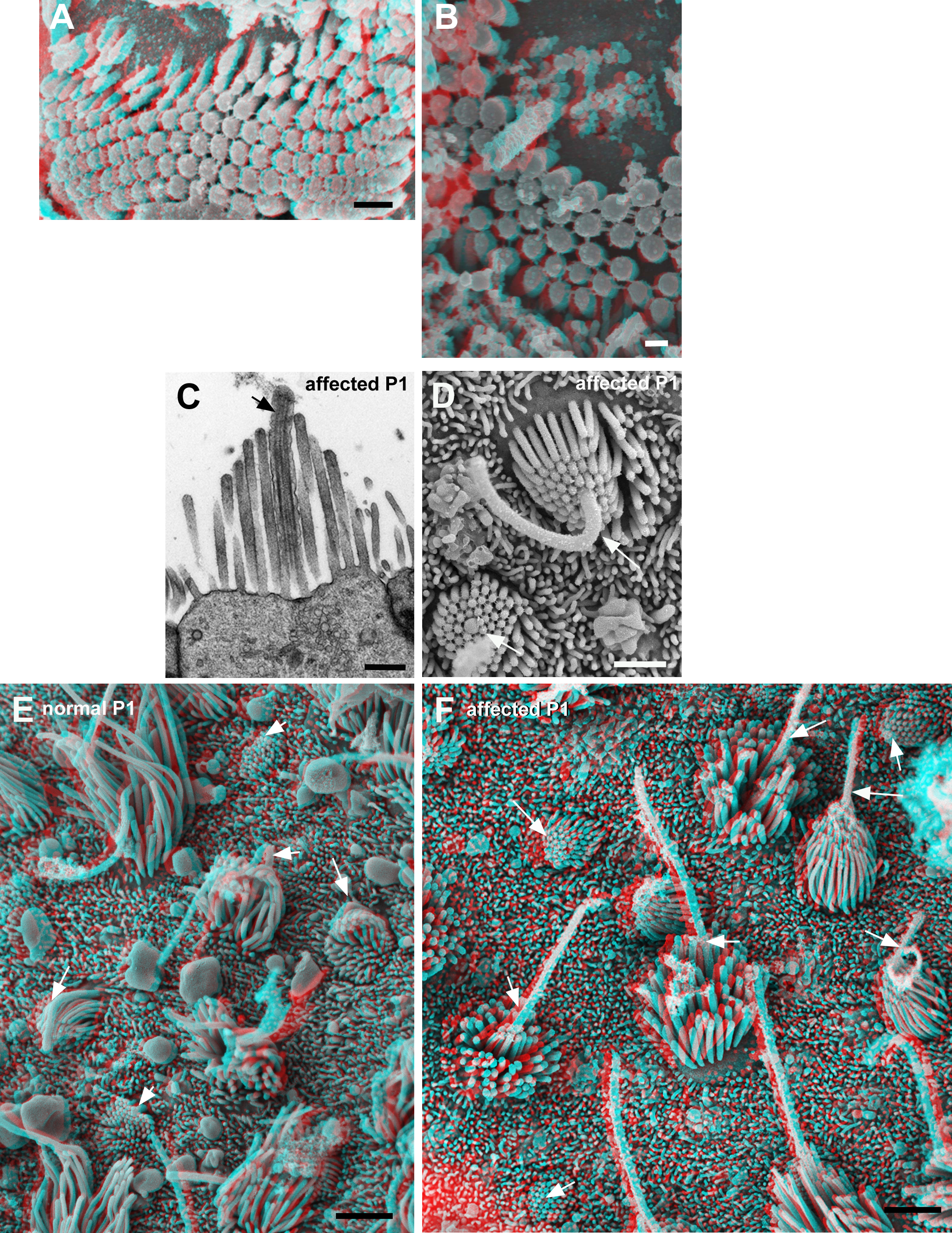

Supplement: S4 Fig — A,B. Anaglyph stereoimages of IHC hair bundles of an affected animal at P6. A. Height gradient across the bundle from inner to outer side. B. Height gradient of stereocilia towards kinocilium located in the centre of the cell surface. Gradient is thus, from periphery to centre, the opposite to that in A. Various cross links between stereocilia are evident. Scale bars: 1μm. C-F Hair bundle maturation in utricular maculae at P1. C. Thin section of hair cell in affected animal at P1. Kinocilium (arrowed) in centre of bundle B. Hair bundles in affected animal at P1. Entire, round apical surface of hair cell covered in stereociia of almost equal height with kinocilium (arrow) emerging from the centre. C, D. Anaglyph stereoimages. In the macula from a normal animal (C) all the kinocilia (arrows) are located eccentrically at one side of the bundle of stereocilia which show progressive stages of maturation on different hair cells. In the affected macula (D) in every bundle from, those with the shortest stereocilia to those with the longest, the kinocilium (arrowed) arises from the centre of the bundle at approximately the centre of the apical surface of the hair cell. Scale bars: A,B 1μm; C,D 2μm. (TIF) [file pgen.1006692.s004.tif]

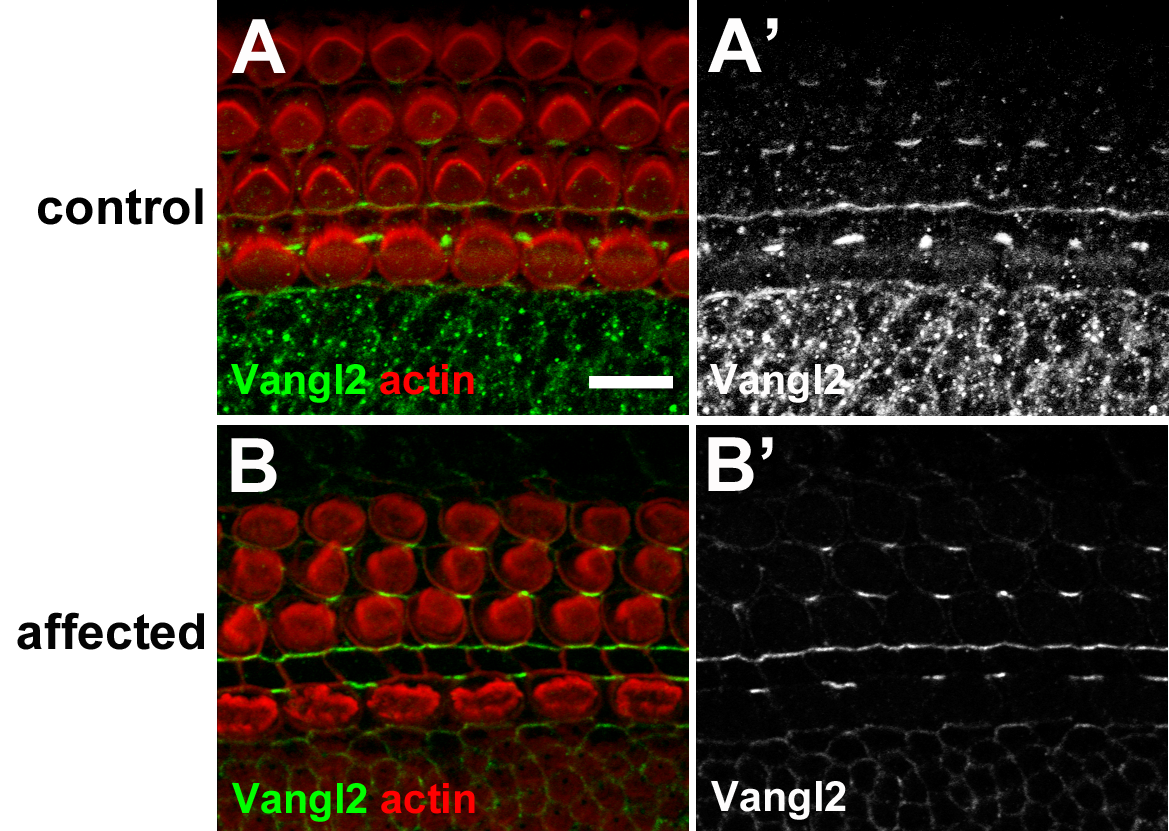

Supplement: S5 Fig — A, in a whole-mount of a normal (control) mouse organ of Corti an anti-Vangl2 antibody labelled apical junctions between supporting cells and hair cells (arrows). B, in an affected littermate the Vangl2 antibody stained comparable structures. Scale bar: 10μm (refers to all panels). (TIF) [file pgen.1006692.s005.tif]
